# Supplementary material for: Environmental DNA from plastic and textile marine litter detects exotic and nuisance species nearby ports
Source: PLoS One. 2020 Jun 18;15(6):e0228811. doi: 10.1371/journal.pone.0228811 (PMC7302909; doi:10.1371/journal.pone.0228811)
Supplement: S1 Table — (DOCX) [file pone.0228811.s001.docx]

|  | **Minimum identity and e value** | | | | |
| --- | --- | --- | --- | --- | --- |
|  | **90%, E-10** | **97%, E-10** | **90%, E-50** | **95%, E-50** | **97%, E-50** |
| **Ñ-P1** | 1642 | 1126 | 1216 | 1198 | 969 |
| **P-T1** | 6629 | 4195 | 2044 | 1406 | 1193 |
| **A-1** | 606 | 404 | 287 | 120 | 81 |
| **P-P1** | 68455 | 45379 | 40688 | 35988 | 30241 |
| **Ñ-P2** | 19279 | 8505 | 6898 | 4189 | 3417 |
| **Ñ-T** | 2399 | 425 | 342 | 127 | 97 |
| **C-P1** | 491 | 205 | 2 | 1 | 0 |
| **P-P2** | 7301 | 2393 | 1087 | 597 | 366 |
| **A-P2** | 6504 | 1612 | 1279 | 179 | 131 |
| **C-P2** | 8159 | 4129 | 2114 | 1949 | 1379 |
| **C-T** | 18421 | 10819 | 4455 | 3921 | 3137 |
| **R-P1** | 17472 | 399 | 154 | 66 | 53 |

**S1 Table**. Number of sequences assigned to a species level with different BLAST conditions. Beach acronyms stand as A, C, Ñ, P and R for Arbeyal, Cagonera, Ñora, Peñarrubia and Rinconin respectively.
