# Supplementary material for: Environmental DNA from plastic and textile marine litter detects exotic and nuisance species nearby ports
Source: PLoS One. 2020 Jun 18;15(6):e0228811. doi: 10.1371/journal.pone.0228811 (PMC7302909; doi:10.1371/journal.pone.0228811)
Supplement: S2 Table — (DOCX) [file pone.0228811.s002.docx]

| **S2 Table.** Number of sequences assigned to each species in the biofilm samples analyzed. | | | | | | | | | | | | |
| --- | --- | --- | --- | --- | --- | --- | --- | --- | --- | --- | --- | --- |
| **Class** | **Species** | **A-P1** | **Ñ-P2** | **P-P2** | **C-T** | **R-P1** | **Ñ-P1** | **C-P2** | **A-P2** | **P-T1** | **Ñ-T** | **P-P1** |
| Actinopterygii | *Labrus bergylta* | 0 | 0 | 0 | 3 | 0 | 0 | 0 | 0 | 0 | 0 | 0 |
| Actinopterygii | *Symphodus melops* | 0 | 0 | 0 | 8 | 0 | 0 | 0 | 0 | 0 | 0 | 0 |
| Aves | *Meleagris gallopavo* | 0 | 0 | 0 | 0 | 0 | 0 | 0 | 7 | 0 | 0 | 0 |
| Bacillariophyceae | *Tabularia* sp. | 0 | 0 | 0 | 0 | 0 | 0 | 0 | 2 | 0 | 0 | 0 |
| Bangiophyceae | *Porphyra umbilicalis* | 0 | 0 | 0 | 0 | 7 | 0 | 0 | 0 | 0 | 0 | 32 |
| Bivalvia | *Mytilus edulis* | 0 | 0 | 0 | 3 | 0 | 0 | 0 | 0 | 0 | 0 | 0 |
| Cephalopoda | *Dosidicus gigas* | 0 | 0 | 0 | 0 | 1 | 0 | 0 | 0 | 0 | 0 | 0 |
| Cephalopoda | *Illex argentinus* | 0 | 0 | 0 | 0 | 0 | 0 | 0 | 0 | 0 | 0 | 15 |
| Cephalopoda | *Sepia officinalis* | 0 | 0 | 0 | 0 | 0 | 0 | 0 | 0 | 0 | 0 | 203 |
| Conoidasida | *Isospora sp.* | 0 | 0 | 1 | 0 | 0 | 0 | 0 | 0 | 0 | 0 | 0 |
| Coscinodiscophyceae | *Podosira stelligera* | 0 | 0 | 0 | 143 | 5 | 0 | 0 | 0 | 26 | 0 | 0 |
| Dothideomycetes | *Cladosporium bruhnei* | 0 | 0 | 14 | 5 | 0 | 0 | 0 | 0 | 0 | 0 | 246 |
| Dothideomycetes | *Cladosporium herbarum* | 0 | 1 | 12 | 0 | 0 | 0 | 0 | 0 | 0 | 0 | 11 |
| Dothideomycetes | *Cladosporium tenuissimum* | 0 | 27 | 40 | 0 | 0 | 0 | 9 | 0 | 4 | 0 | 0 |
| Dinophyceae | *Alexandrium affine* | 0 | 0 | 0 | 0 | 0 | 0 | 2 | 0 | 0 | 0 | 0 |
| Dinophyceae | *Alexandrium catenella* | 0 | 0 | 0 | 0 | 0 | 0 | 0 | 0 | 0 | 0 | 1 |
| Dinophyceae | *Alexandrium minutum* | 0 | 0 | 0 | 1 | 0 | 0 | 3 | 0 | 0 | 0 | 0 |
| Dinophyceae | *Alexandrium ostenfeldii* | 0 | 0 | 0 | 1 | 0 | 0 | 0 | 0 | 0 | 0 | 0 |
| Dinophyceae | *Alexandrium sp.* | 0 | 0 | 0 | 0 | 0 | 0 | 0 | 0 | 0 | 0 | 1 |
| Dinophyceae | *Azadinium poporum* | 0 | 0 | 0 | 2 | 0 | 0 | 0 | 0 | 0 | 0 | 0 |
| Dinophyceae | *Cryptoperidiniopsis sp.* | 0 | 0 | 0 | 0 | 0 | 0 | 0 | 0 | 0 | 0 | 30 |
| Dinophyceae | *Karenia brevis* | 0 | 0 | 0 | 1 | 0 | 0 | 0 | 0 | 0 | 0 | 0 |
| Dinophyceae | *Karlodinium sp.* | 0 | 0 | 0 | 1 | 0 | 0 | 0 | 0 | 0 | 0 | 0 |
| Dinophyceae | *Lepidodinium chlorophorum* | 0 | 0 | 0 | 1 | 0 | 0 | 0 | 0 | 0 | 0 | 0 |
| Dinophyceae | *Peridinium inconspicuum* | 0 | 0 | 0 | 0 | 0 | 0 | 0 | 0 | 0 | 0 | 9 |
| Dinophyceae | *Peridinium* sp. | 0 | 0 | 0 | 0 | 0 | 0 | 0 | 0 | 0 | 0 | 1 |
| Dinophyceae | *Peridinium sp.* | 0 | 0 | 0 | 0 | 0 | 0 | 0 | 0 | 0 | 0 | 1 |
| Dinophyceae | *Prorocentrum micans* | 0 | 0 | 0 | 0 | 0 | 0 | 0 | 0 | 0 | 0 | 2 |
| Dinophyceae | *Prorocentrum sp.* | 0 | 0 | 0 | 0 | 0 | 0 | 0 | 0 | 0 | 0 | 2 |
| Dinophyceae | *Scrippsiella lachrymosa* | 0 | 0 | 0 | 0 | 0 | 0 | 0 | 0 | 0 | 0 | 1 |
| Dinophyceae | *Scrippsiella sp.* | 0 | 0 | 0 | 4 | 0 | 0 | 0 | 0 | 0 | 0 | 12 |
| Dinophyceae | *Scrippsiella sp.* | 0 | 0 | 0 | 0 | 0 | 0 | 0 | 0 | 0 | 0 | 14 |
| Dinophyceae | *Symbiodinium sp.* | 0 | 0 | 0 | 1 | 0 | 0 | 0 | 0 | 0 | 0 | 0 |
| Dinophyceae | *Thoracosphaera heimii* | 0 | 0 | 0 | 1 | 0 | 0 | 0 | 0 | 0 | 0 | 125 |
| Dinophyceae | *Woloszynskia sp.* | 0 | 0 | 0 | 1 | 0 | 0 | 0 | 0 | 0 | 0 | 0 |
| Echinoidea | *Paracentrotus lividus* | 0 | 0 | 0 | 5 | 0 | 0 | 0 | 0 | 0 | 0 | 0 |
| Entognatha | *Hypogastruridae sp.* | 0 | 60 | 0 | 0 | 0 | 0 | 0 | 0 | 0 | 0 | 0 |
| Eurotiomycetes | *Eupenicillium crustaceum* | 0 | 0 | 0 | 0 | 0 | 0 | 0 | 0 | 0 | 0 | 1 |
| Eurotiomycetes | *Penicillium albocoremium* | 0 | 0 | 0 | 0 | 0 | 1 | 3 | 0 | 0 | 0 | 0 |
| Eurotiomycetes | *Penicillium bialowiezense* | 0 | 0 | 0 | 0 | 0 | 0 | 0 | 0 | 0 | 0 | 12 |
| Eurotiomycetes | *Penicillium brevicompactum* | 0 | 4 | 0 | 0 | 0 | 0 | 0 | 0 | 0 | 0 | 5 |
| Eurotiomycetes | *Penicillium carneum* | 0 | 0 | 0 | 3 | 0 | 0 | 0 | 0 | 0 | 0 | 0 |
| Eurotiomycetes | *Penicillium chrysogenum* | 0 | 51 | 0 | 2 | 0 | 0 | 0 | 0 | 0 | 0 | 0 |
| Eurotiomycetes | *Penicillium clavigerum* | 0 | 0 | 0 | 0 | 0 | 0 | 0 | 0 | 0 | 0 | 11 |
| Eurotiomycetes | *Penicillium digitatum* | 0 | 0 | 0 | 3 | 0 | 0 | 0 | 0 | 0 | 0 | 0 |
| Eurotiomycetes | *Penicillium dipodomyis* | 0 | 5 | 0 | 0 | 0 | 0 | 0 | 0 | 0 | 0 | 2 |
| Eurotiomycetes | *Penicillium italicum* | 0 | 1 | 0 | 5 | 0 | 0 | 0 | 0 | 0 | 0 | 4 |
| Eurotiomycetes | *Penicillium persicinum* | 0 | 3 | 0 | 0 | 0 | 0 | 0 | 0 | 0 | 0 | 2 |
| Eurotiomycetes | *Penicillium polonicum* | 0 | 0 | 0 | 1 | 0 | 0 | 3 | 0 | 0 | 0 | 0 |
| Eurotiomycetes | *Penicillium roqueforti* | 0 | 0 | 0 | 0 | 0 | 0 | 0 | 0 | 0 | 0 | 25 |
| Eurotiomycetes | *Penicillium soppii* | 0 | 0 | 0 | 1 | 0 | 0 | 0 | 0 | 0 | 0 | 0 |
| Eurotiomycetes | *Penicillium venetum* | 0 | 0 | 0 | 0 | 0 | 0 | 4 | 0 | 0 | 0 | 0 |
| Florideophyceae | *Colaconema sp.* | 0 | 0 | 0 | 0 | 0 | 0 | 0 | 0 | 0 | 3 | 0 |
| Florideophyceae | *Corallina ferreyrae* | 0 | 0 | 0 | 2 | 0 | 0 | 76 | 0 | 13 | 41 | 0 |
| Florideophyceae | *Corallina officinalis* | 0 | 0 | 0 | 0 | 0 | 0 | 22 | 0 | 2 | 5 | 0 |
| Florideophyceae | *Corallina sp.* | 0 | 0 | 0 | 0 | 0 | 0 | 38 | 0 | 14 | 15 | 0 |
| Florideophyceae | *Gelidium spinosum* | 0 | 0 | 0 | 0 | 0 | 0 | 38 | 0 | 2 | 0 | 0 |
| Florideophyceae | *Helminthocladia calvadosii* | 0 | 0 | 0 | 0 | 0 | 0 | 57 | 0 | 0 | 0 | 0 |
| Florideophyceae | *Meredithia microphylla* | 0 | 0 | 0 | 1 | 0 | 0 | 0 | 0 | 0 | 0 | 0 |
| Florideophyceae | *Phyllophora sp*. | 0 | 0 | 0 | 4 | 0 | 0 | 0 | 0 | 0 | 0 | 0 |
| Florideophyceae | *Plocamium cartilagineum* | 0 | 0 | 0 | 7 | 0 | 0 | 0 | 0 | 0 | 0 | 0 |
| Florideophyceae | *Plocamium raphelisianum* | 0 | 0 | 0 | 0 | 0 | 0 | 0 | 0 | 27 | 0 | 0 |
| Florideophyceae | *Plocamium sp.* | 0 | 0 | 0 | 1 | 0 | 0 | 0 | 0 | 0 | 0 | 0 |
| Gastropoda | *Patella vulgata* | 0 | 0 | 0 | 0 | 1 | 0 | 0 | 0 | 1 | 0 | 0 |
| Gastropoda | *Tricolia pullus* | 0 | 0 | 0 | 1 | 0 | 0 | 0 | 0 | 0 | 0 | 0 |
| Gymnolaemata | *Celleporella* sp. | 0 | 0 | 0 | 0 | 0 | 0 | 0 | 0 | 4 | 5 | 0 |
| Gymnolaemata | *Electra pilosa* | 0 | 0 | 0 | 3 | 0 | 0 | 0 | 3 | 5 | 3 | 0 |
| Hexanauplia | *Clausocalanus arcuicornis* | 0 | 0 | 0 | 1 | 0 | 0 | 0 | 0 | 1 | 0 | 0 |
| Hexanauplia | *Clausocalanus jobei* | 0 | 0 | 0 | 6 | 0 | 0 | 0 | 0 | 0 | 0 | 0 |
| Hexanauplia | *Clausocalanus pergens* | 0 | 0 | 0 | 19 | 0 | 0 | 0 | 0 | 0 | 0 | 0 |
| Hexanauplia | *Ctenocalanus vanus* | 0 | 0 | 0 | 2 | 0 | 0 | 0 | 0 | 0 | 0 | 0 |
| Hexanauplia | *Cyclopoida sp.* | 0 | 16 | 1 | 0 | 0 | 11 | 7 | 1 | 0 | 0 | 95 |
| Hexanauplia | *Paracalanus parvus* | 0 | 0 | 0 | 0 | 0 | 0 | 0 | 0 | 0 | 0 | 271 |
| Hexanauplia | *Pseudocalanus elongatus* | 0 | 0 | 0 | 11 | 0 | 0 | 0 | 0 | 0 | 0 | 0 |
| Hydrozoa | *Campanularia hincksii* | 0 | 0 | 0 | 10 | 0 | 0 | 0 | 0 | 0 | 0 | 0 |
| Hydrozoa | *Clytia gracilis* | 0 | 0 | 0 | 4 | 0 | 0 | 0 | 0 | 0 | 0 | 0 |
| Hydrozoa | *Clytia paulensis* | 0 | 0 | 0 | 80 | 1 | 0 | 0 | 0 | 0 | 0 | 58 |
| Hydrozoa | *Muggiaea atlantica* | 0 | 0 | 0 | 1 | 0 | 0 | 0 | 0 | 0 | 0 | 0 |
| Insecta | *Baetis rhodani* | 0 | 207 | 0 | 0 | 0 | 0 | 11 | 0 | 0 | 0 | 0 |
| Insecta | *Culex bahamensis* | 0 | 1 | 0 | 0 | 0 | 1 | 0 | 0 | 1 | 0 | 1 |
| Insecta | Psocoptera sp. | 0 | 0 | 0 | 0 | 0 | 0 | 1 | 0 | 0 | 0 | 0 |
| Insecta | *Psychoda grisescens* | 0 | 95 | 0 | 0 | 0 | 0 | 0 | 0 | 0 | 0 | 0 |
| Malacostraca | *Gammarus crinicaudatus* | 0 | 0 | 0 | 0 | 0 | 0 | 0 | 0 | 0 | 0 | 1 |
| Malacostraca | *Pacifastacus leniusculus* | 0 | 0 | 0 | 0 | 0 | 6 | 0 | 0 | 0 | 0 | 0 |
| Mammalia | *Bos taurus* | 0 | 0 | 0 | 0 | 0 | 0 | 8 | 0 | 0 | 0 | 0 |
| Mammalia | *Homo sapiens* | 0 | 2799 | 296 | 2304 | 35 | 944 | 459 | 52 | 1075 | 25 | 28972 |
| Mammalia | *Sus scrofa* | 0 | 0 | 0 | 0 | 0 | 0 | 5 | 0 | 0 | 0 | 0 |
| Ophiuroidea | *Ophiothrix sp.* | 0 | 0 | 0 | 5 | 0 | 0 | 0 | 0 | 0 | 0 | 0 |
| Polychaeta | *Dasybranchus sp.* | 0 | 0 | 0 | 0 | 0 | 0 | 0 | 0 | 0 | 0 | 3 |
| Polychaeta | *Platynereis dumerilii* | 0 | 0 | 0 | 0 | 0 | 0 | 0 | 0 | 4 | 0 | 0 |
| Polychaeta | *Syllidia armata* | 0 | 0 | 0 | 359 | 0 | 0 | 0 | 0 | 0 | 0 | 0 |
| Sordariomycetes | *Cordyceps confragosa* | 0 | 0 | 0 | 0 | 0 | 0 | 0 | 0 | 0 | 0 | 6 |
| Sordariomycetes | *Emericellopsis minima* | 0 | 0 | 0 | 3 | 0 | 0 | 0 | 0 | 0 | 0 | 0 |
| Sordariomycetes | *Fusarium solani* | 0 | 0 | 0 | 4 | 0 | 0 | 0 | 0 | 0 | 0 | 0 |
| Sordariomycetes | *Lecanicillium muscarium* | 0 | 0 | 0 | 0 | 0 | 0 | 0 | 0 | 0 | 0 | 1 |
| Sordariomycetes | *Metarhizium anisopliae* | 0 | 0 | 1 | 0 | 0 | 0 | 0 | 0 | 0 | 0 | 0 |
