# Supplementary material for: Environmental DNA from plastic and textile marine litter detects exotic and nuisance species nearby ports
Source: PLoS One. 2020 Jun 18;15(6):e0228811. doi: 10.1371/journal.pone.0228811 (PMC7302909; doi:10.1371/journal.pone.0228811)
Supplement: S3 Table — (DOCX) [file pone.0228811.s003.docx]

| **Class** | **Species** | **Gijon Port** | **Arbeyal** | **Rinconin** | **Peñarrubia** | **Cagonera** | **Ñora** |
| --- | --- | --- | --- | --- | --- | --- | --- |
| Polychaeta | Dasybranchus sp. | 0 | 0 | 0 | 1 | 0 | 0 |
| Polychaeta | *Leodice harassii* | 1 | 0 | 0 | 0 | 0 | 0 |
| Polychaeta | *Syllidia armata* | 0 | 0 | 0 | 0 | 1 | 0 |
| Polychaeta | *Platynereis dumerilii* | 13 | 0 | 0 | 1 | 0 | 0 |
| Polychaeta | *Nereis falsa* | 1 | 0 | 0 | 0 | 0 | 0 |
| Polychaeta | *Sabellaria spinulosa* | 0 | 0 | 1 | 0 | 0 | 0 |
| Polychaeta | *Spirobranchus latiscapus* | 0 | 0 | 0 | 0 | 1 | 0 |
| Polychaeta | *Syllis gracilis* | 1 | 0 | 0 | 0 | 0 | 0 |
| Hexanauplia | *Perforatus perforatus* | 2 | 0 | 0 | 0 | 1 | 0 |
| Malacostraca | *Ampithoe rubricata* | 1 | 0 | 0 | 0 | 0 | 0 |
| Malacostraca | *Pacifastacus leniusculus* | 0 | 0 | 0 | 0 | 0 | 1 |
| Malacostraca | *Livoneca redmanii* | 5 | 0 | 0 | 0 | 0 | 0 |
| Malacostraca | *Gammarus crinicaudatus* | 0 | 0 | 0 | 1 | 0 | 0 |
| Malacostraca | Palaemon sp | 4 | 0 | 0 | 0 | 0 | 0 |
| Malacostraca | *Pilumnus hirtellus* | 1 | 0 | 0 | 0 | 0 | 0 |
| Gymnolaemata | *Electra pilosa* | 0 | 1 | 0 | 1 | 1 | 1 |
| Gymnolaemata | Celleporella sp | 0 | 0 | 0 | 1 | 0 | 1 |
| Gymnolaemata | *Amathia verticillata* | 7 | 0 | 0 | 0 | 0 | 0 |
| Gymnolaemata | *Watersipora subtorquata* | 3 | 0 | 0 | 0 | 0 | 0 |
| Ascidiacea | *Morchellium argus* | 1 | 0 | 0 | 0 | 0 | 0 |
| Ascidiacea | *Microcosmus squamiger* | 1 | 0 | 0 | 0 | 0 | 0 |
| Ascidiacea | *Botryllus schlosseri* | 1 | 0 | 0 | 0 | 0 | 0 |
| Ascidiacea | *Styela plicata* | 3 | 0 | 0 | 0 | 0 | 0 |
| Hydrozoa | *Campanularia hincksii* | 0 | 0 | 0 | 0 | 1 | 0 |
| Hydrozoa | *Clytia gracilis* | 0 | 0 | 0 | 0 | 1 | 0 |
| Hydrozoa | *Clytia paulensis* | 0 | 0 | 1 | 1 | 1 | 0 |
| Hydrozoa | *Muggiaea atlantica* | 0 | 0 | 0 | 0 | 1 | 0 |
| Echinoidea | *Paracentrotus lividus* | 0 | 0 | 0 | 0 | 1 | 0 |
| Ophiuroidea | Ophiothrix sp. | 0 | 0 | 0 | 0 | 1 | 0 |
| Bivalvia | *Neopycnodonte cochlear* | 0 | 0 | 0 | 1 | 0 | 0 |
| Bivalvia | *Mytilaster minimus* | 11 | 0 | 0 | 0 | 0 | 0 |
| Bivalvia | Mytilus sp | 32 | 0 | 0 | 0 | 1 | 0 |
| Gastropoda | *Felimare villafranca* | 2 | 0 | 0 | 0 | 0 | 0 |
| Gastropoda | *Peringia ulvae* | 0 | 1 | 0 | 0 | 0 | 0 |
| Gastropoda | Patella sp | 9 | 0 | 1 | 1 | 0 | 0 |
| Gastropoda | *Tricolia pullus* | 0 | 0 | 0 | 0 | 1 | 0 |
| Demospongiae | *Pachymatisma johnstonia* | 0 | 0 | 0 | 0 | 1 | 0 |
| Demospongiae | *Pachymatisma normani* | 0 | 0 | 0 | 0 | 1 | 0 |
| Demospongiae | *Oscarella lobularis* | 0 | 0 | 0 | 0 | 1 | 1 |

**S3 Table**. Macroscopic animal species found from conventional sampling in Gijon port by Miralles et al. 2016 [42] and from eDNA and NGS on litter biofilms from beaches.
