# Supplementary material for: Environmental DNA from plastic and textile marine litter detects exotic and nuisance species nearby ports
Source: PLoS One. 2020 Jun 18;15(6):e0228811. doi: 10.1371/journal.pone.0228811 (PMC7302909; doi:10.1371/journal.pone.0228811)
Supplement: Attachment — Submitted filename: PONE-D-19-21907_reviewer.pdf [file pone.0228811.s004.pdf]

PLOS ONE

Plastic and textile marine litter as reservoirs for secondary species dispersal from ports

--Manuscript Draft--

**Manuscript Number:**	PONE-D-19-21907
**Article Type:**	Research Article
**Full Title:**	Plastic and textile marine litter as reservoirs for secondary species dispersal from ports
**Short Title:**	Plastic and textile marine litter as reservoirs for secondary species dispersal
**Corresponding Author:**	Aitor Ibabe Arrieta, M.D<br>Universidad de Oviedo Facultad de Medicina y Ciencias de la Salud<br>Oviedo, SPAIN
**Keywords:**	Marine debris; Biofilm; Next Generation Sequencing; Nonindigenous species
**Abstract:**	Marine debris is nowadays an important source of environmental and economic problems. Floating litter can be employed by marine organisms as a surface to attach and use as spreading vector. In this way, human activities are promoting expansions of potentially harmful species into novel ecosystems, putting in danger the autochthonous communities. In this project, more than 1,000 litter items were collected and classified from five beaches eastwards the port of Gijon, in Asturias, Spain. Next generation sequencing was employed to study the communities occurring in biofilm attached to items of different materials. A dominance of DNA from Florideophyceans, Dinophyceans and Arthropods was found, and four non-indigenous species were identified. Results showed a clear preference of Florideophyceans and Bryozoans to attach on textile surfaces versus plastic ones. Considering that these taxa contain several highly invasive species described to date, these data emphasize the potential of textile marine debris as a vector for dispersal of alien species. Moreover, the litter macrofauna profile was more similar with port's macrofauna in closer beaches than in farther ones, confirming that both plastic and textile marine litter can be a vector for species dispersal from ports.
**Order of Authors:**	Aitor Ibabe Arrieta, M.D
	Fernando Rayón
	Jose Luis Martinez
	Eva Garcia-Vazquez
**Additional Information:**	
**Question**	**Response**
**Financial Disclosure**<br><br>Enter a financial disclosure statement that describes the sources of funding for the work included in this submission. Review the submission guidelines for detailed requirements. View published research articles from *PLOS ONE* for specific examples.<br><br>This statement is required for submission and **will appear in the published article** if the submission is accepted. Please make sure it is accurate.	This study was funded by the Spanish Ministry of Economy and Competitiveness, Grant CGL-2016-79209-R.

Powered by Editorial Manager® and ProduXion Manager® from Aries Systems Corporation

**Unfunded studies**<br>Enter: *The author(s) received no specific funding for this work.*<br><br>**Funded studies**<br>Enter a statement with the following details:<br>• Initials of the authors who received each award<br>• Grant numbers awarded to each author<br>• The full name of each funder<br>• URL of each funder website<br>• Did the sponsors or funders play any role in the study design, data collection and analysis, decision to publish, or preparation of the manuscript?<br>• **NO** - Include this sentence at the end of your statement: *The funders had no role in study design, data collection and analysis, decision to publish, or preparation of the manuscript.*<br>• **YES** - Specify the role(s) played.<br><br>* typeset	
**Competing Interests**<br><br>Use the instructions below to enter a competing interest statement for this submission. On behalf of all authors, disclose any competing interests that could be perceived to bias this work—acknowledging all financial support and any other relevant financial or non-financial competing interests.<br><br>This statement **will appear in the published article** if the submission is accepted. Please make sure it is accurate. View published research articles from *PLOS ONE* for specific examples.	The authors have declared that no competing interests exist.

**NO authors have competing interests**<br><br>Enter: *The authors have declared that no competing interests exist.*<br><br>**Authors with competing interests**<br><br>Enter competing interest details beginning with this statement:<br><br>*I have read the journal's policy and the authors of this manuscript have the following competing interests: [insert competing interests here]*<br><br>* typeset	
**Ethics Statement**<br><br>Enter an ethics statement for this submission. This statement is required if the study involved:<br><br>• Human participants<br>• Human specimens or tissue<br>• Vertebrate animals or cephalopods<br>• Vertebrate embryos or tissues<br>• Field research<br><br>Write "N/A" if the submission does not require an ethics statement.<br><br>General guidance is provided below. Consult the submission guidelines for detailed instructions. **Make sure that all information entered here is included in the Methods section of the manuscript.**	N/A

Powered by Editorial Manager® and ProduXion Manager® from Aries Systems Corporation

**Format for specific study types**<br><br>**Human Subject Research (involving human participants and/or tissue)**<br>• Give the name of the institutional review board or ethics committee that approved the study<br>• Include the approval number and/or a statement indicating approval of this research<br>• Indicate the form of consent obtained (written/oral) or the reason that consent was not obtained (e.g. the data were analyzed anonymously)<br><br>**Animal Research (involving vertebrate animals, embryos or tissues)**<br>• Provide the name of the Institutional Animal Care and Use Committee (IACUC) or other relevant ethics board that reviewed the study protocol, and indicate whether they approved this research or granted a formal waiver of ethical approval<br>• Include an approval number if one was obtained<br>• If the study involved *non-human primates*, add *additional details* about animal welfare and steps taken to ameliorate suffering<br>• If anesthesia, euthanasia, or any kind of animal sacrifice is part of the study, include briefly which substances and/or methods were applied<br><br>**Field Research**<br><br>Include the following details if this study involves the collection of plant, animal, or other materials from a natural setting:<br>• Field permit number<br>• Name of the institution or relevant body that granted permission	
**Data Availability**<br><br>Authors are required to make all data underlying the findings described fully available, without restriction, and from the time of publication. PLOS allows rare exceptions to address legal and ethical concerns. See the PLOS Data Policy and FAQ for detailed information.	Yes - all data are fully available without restriction

A Data Availability Statement describing where the data can be found is required at submission. Your answers to this question constitute the Data Availability Statement and **will be published in the article**, if accepted.<br><br>**Important:** Stating 'data available on request from the author' is not sufficient. If your data are only available upon request, select 'No' for the first question and explain your exceptional situation in the text box.<br><br>Do the authors confirm that all data underlying the findings described in their manuscript are fully available without restriction?	
**Describe where the data may be found in full sentences. If you are copying our sample text, replace any instances of XXX with the appropriate details.**<br><br>• If the data are **held or will be held in a public repository**, include URLs, accession numbers or DOIs. If this information will only be available after acceptance, indicate this by ticking the box below. For example: *All XXX files are available from the XXX database (accession number(s) XXX, XXX.)*.<br>• If the data are all contained **within the manuscript and/or Supporting Information files**, enter the following: *All relevant data are within the manuscript and its Supporting Information files.*<br>• If neither of these applies but you are able to provide **details of access elsewhere**, with or without limitations, please do so. For example:<br><br>*Data cannot be shared publicly because of [XXX]. Data are available from the XXX Institutional Data Access / Ethics Committee (contact via XXX) for researchers who meet the criteria for access to confidential data.*<br><br>*The data underlying the results presented in the study are available from (include the name of the third party*	All relevant data are within the manuscript and its Supporting Information files.

and contact information or URL).<br>• This text is appropriate if the data are owned by a third party and authors do not have permission to share the data.<br><br>* typeset	
Additional data availability information:	Tick here if the URLs/accession numbers/DOIs will be available only after acceptance of the manuscript for publication so that we can ensure their inclusion before publication.

Powered by Editorial Manager® and ProduXion Manager® from Aries Systems Corporation

Manuscript Click here to download Manuscript Manuscript.docx

Plastic and textile marine litter as reservoirs for secondary species dispersal from ports.

Aitor Ibabe*, Fernando Rayón, Jose Luis Martinez, Eva Garcia-Vazquez.

*Corresponding author
E-mail: ibabeaitor@gmail.com

Address: Department of Functional Biology, University of Oviedo. C/ Julian Claveria s/n. 33006-Oviedo, Spain.

Abstract

Marine debris is nowadays an important source of environmental and economic problems. Floating litter can be employed by marine organisms as a surface to attach and use as spreading vector. In this way, human activities are promoting expansions of potentially harmful species into novel ecosystems, putting in danger the autochthonous communities. In this project, more than 1,000 litter items were collected and classified from five beaches eastwards the port of Gijon, in Asturias, Spain. Next generation sequencing was employed to study the communities occurring in biofilm attached to items of different materials. A dominance of DNA from Florideophyceans, Dinophyceans and Arthropods was found, and four non-indigenous species were identified. Results showed a clear preference of Florideophyceans and Bryozoans to attach on textile surfaces versus plastic ones. Considering that these taxa contain several highly invasive species described to date, these data emphasize the potential of textile marine debris as a vector for dispersal of alien species. Moreover, the litter macrofauna profile was more similar with port's macrofauna in closer beaches than in farther ones, confirming that both plastic and textile marine litter can be a vector for species dispersal from ports.

Keywords: Marine debris; Biofilm; Next generation sequencing; Nonindigenous species

Introduction

Human activities are triggering environmental changes all over the world since the beginning of intensive production methods. Activities such as agriculture, fisheries or industry are overexploiting the natural resources and this has led to a situation where species extinction rates have become 100 to 1000 times higher than the ones before human domination [1]. A huge amount of the waste produced from this excessive human activity is ending up in the ocean, altering marine ecosystems. These materials are known as marine debris or marine litter. This problem has led to a difficult situation, not only for the conservation of marine ecosystems, but also for human health and economic activities. Plastic litter that is floating in the oceans is an important cause of mortality for many animals such as marine mammals, seabirds or turtles, either because they ingest it [2-3] and/or get entangled [4-6]. In addition, marine litter causes important economic losses in industries such as fisheries, because of the time spent cleaning the debris from nets, and net losses. As an example, marine plastics cost an average of between $15 million and $17 million per year to the Scottish fishing industry [7]. Tourism can also suffer negative impacts due to the presence of marine litter on the coasts, which can affect the public perception of the quality of the surrounding environment leading to a loss of income for this sector [8].

Regarding other types of potential threats caused by marine debris, its role as a dispersal vector by invasive organisms is a fact of special concern [9]. Marine litter promotes the establishment and dispersal of non-native species. It can provide surface for colonizing species, facilitating their spread to new habitats [10]. Newly entered colonizers can alter the local ecosystem affecting the native organisms in several ways, from competition with or predation on native species to habitat alteration to transmission of exotic diseases to local species [11-13]. In addition to the impacts on local biodiversity, non-indigenous species have also severe impacts on the economy. For example, in the United States, more than $138 billion per year are used to control new colonizers, or to avoid infections of non-indigenous diseases [14]. Aquaculture industries are also affected by invasive species that can alter the productivity, as in the case of *Caulerpa taxifolia* which forms dense mats [15], or *Carcinus maenas* which consumes native commercially important clams in Tasmania [16].

Identifying the biota that arrives in the local ecosystem is the only way to detect alien species and control invasions. However, quite often invaders are spread in an early ontogenetic stage (e.g. eggs, larvae or algae propagules) and they are not visually identifiable, thus non-indigenous individuals may remain undetected until they are already adults and start reproducing and expanding [17-18]. Exhaustive monitoring is needed, with low probabilities of finding non-stablished alien species due to their low density [19-20]. Identification based on organism morphology requires expert taxonomists specialized on the taxa to be analyzed, and often (especially in early development stages) identification cannot be done to a species level, limiting it to higher groups such as genus or family, which would not be useful for non-indigenous species identifications [21].

Nowadays, new techniques have been developed and species identification can be done based on sequencing and analysing nucleic acids extracted from environmental samples [22], also called environmental DNA or RNA (eDNA, eRNA). Metabarcoding is a well-established method for the detection of non-indigenous species and for biosecurity applications [23-26]. In fact, techniques based on eDNA are advantageous when detecting species with low densities (such as alien species at their arrival and before establishing), as very low DNA concentrations may be enough to find a species when the individuals are still very scarce and/or small [27-28].

Predicting invasions requires understanding the process of the invasion [29-30]; it is therefore crucial to understand how marine debris is spread, and to study the organisms with the capacity of attaching to these surfaces. There are solid studies about marine plastics and their capacity to carry invasive species [31-38]; however, to our knowledge no studies have been done some types of litter surfaces, for example textile litter, as a vector. Ports being identified as potential donors of both marine litter and invasive species studies of biota dispersing on different types of marine litter near ports seem to be necessary to predict how much invasive biota can be dispersed using this vector.

In this study, biota attached to litter items of different materials was characterized using next generation sequencing on DNA extracted from the biofilm, to analyze the composition of the communities inhabiting the marine debris. The biofilm of about 1.5% of surface of the litter objects collected from beaches within 20 km distance from Port of Gijon (central south Bay of Biscay, Spain), where several exotic and invasive

species have been reported [39], was sampled. The relationship between the fauna inventoried in the port and the one found on litter objects nearby was explored.

Material and methods

Sampling

Five beaches located at the east of Gijon port (central south Bay of Biscay) were selected for litter sampling: Arbeyal, El Rinconin, Peñarrubia, Cagonera and La Ñora (Fig. 1). Being Gijón port a potential donor of marine invasive species, the reason for this location is that the dominant currents go eastwards in this coast during the winter [40], that was the sampling time of this study.

Fig. 1 Map showing the location in the northern Spanish coast of the five beaches sampled

From the 13th to the 17th of January 2017, litter items were collected from the five beaches. Sampling was carried out around the lowest diurnal tide (starting 2 hours before and ending 2 hours after) in order to increase the beach surface available to sample. All pieces bigger than 5 cm^2 were collected and their surface was estimated. They were classified in situ in different types: sanitary pads, textiles, plastic bags, plastic bottles, expanded polystyrene (EPS) fragments, fishing gear, and others. Immediately after classification, the items or item fragments representing approximately 0.25% of the total litter surface, representative of the litter profile in each beach, were collected and stored in ethanol for further biofilm sampling and extraction of environmental DNA.

Taxonomy

For the names of the species we followed the taxonomic nomenclature from the World Register of Marine Species [41]. Regarding the status of the species detected visually

and from DNA, exotic and invasive species were identified from the European Network on Invasive Alien Species database NOBANIS [42].

Environmental DNA extraction and metabarcoding

Sterile swabs and gauzes were employed to take out the attached biofilm from the litter by scratching the surface. Sterile DNA/RNA free distilled water was used to rinse and clean the surface. After the biofilm was recovered from the litter, the cotton extremes of the swabs were cut and collected with the gauzes in 15ml Falcon tubes with the water that was also employed to take out the biofilm. Then they were macerated for 2 minutes using a Stomacher 80 biomaster (Seward, UK). A negative control was prepared for this whole procedure, by using sterile swab and gauze extremes and suspending them into Sterile DNA/RNA free distilled water. Once the Stomacher finished, liquid excess was squeezed from the rests of swabs and gauzes and the suspension was pelleted by centrifugation (3000 x g 15 min) following the procedure reported by Pochon et al. (2015) [43]. The supernatant was discarded and then DNA was extracted from the pellet using an E.Z.N.A® Soil DNA Kit (Omega Bio-tek, USA) following the manufacturer's instructions.

The primers mICOIintF and jgHCO2198 [44] were employed to amplify a fragment of ≈300bp within the COI gene (miniCOI). Both primers were modified to include the specific sequences needed for Ion PGM libraries. A single common forward primer was used. Reverse primers were modified to include barcodes for each of the samples, so that 16 different barcoded reverse primers were used. Each barcode has a known sequence to identify the samples after the whole process. Before sequencing, the quantity and quality of the DNA from PCR products was measured using Bioanalyzer (Agilent technologies). The PCR reactions were performed using negative controls to monitor possible contamination. Thermocycling conditions were: 1x: 95°C for 5 min; 35x: 95°C for 1 min, 48°C for 1 min and 72°C for 1 min; 1x: 72°C for 5 min and 4°C on hold. The amplicons were analysed directly in the platform Ion Torrent PGM (ThermoFisher Scientific, USA), in the Unit of DNA Analysis of the Scientific & Technical Services of the University of Oviedo.

Bioinformatics pipeline for analysis of NGS data

Bioinformatics analyses were performed using QIIME, an open-source bioinformatics pipeline [45]. Firstly, an initial screening was carried out in order to select reliable sequences, with a quality value > 20 and a length >200 bp. This was performed using the script split_libraries.py. For taxonomic assignment, the following script was used: assign_taxonomy.py. Instead of using the whole GenBank as a reference, a specific database containing only eukaryotic COI sequences was generated with the script entrez.qiime (Chris Baker. ccmbaker@fas.harvard.edu. Pierce Lab, Department of Organismic and Evolutionary Biology, Harvard University). An initial assignment was made considering a minimum identity of 97% and an E-value of $1e^{-10}$, as it was considered enough to obtain reliable species identification from COI barcodes [46]. In addition, assignments were also done employing minimum identity of 95% and E-value of $1e^{-50}$, to compare results. From the operational taxonomic unit (OTU) table obtained after the assignment, only marine and brackish taxa were retained for further statistical analysis.

A subset of 50 sequences assigned to a species level from each parameter set were randomly taken from the OTU table. They were assigned manually against GenBank using NCBI´s BLAST web browser at https://blast.ncbi.nlm.nih.gov/Blast.cgi?PAGE_TYPE=BlastSearch (NCBI webpage, accessed July 2019), for double-checking the reliability of the taxonomic identification.

Statistical analyses

The statistical analysis was carried out with parametric or non-parametric tests done in PAST program [47] after checking normality in the dataset. For beach litter composition, the proportion of each type of debris was compared among beaches using non-parametric contingency Chi-square, confirmed from Monte Carlo procedure (n=9999 permutations). The litter composition was compared between pairs of beaches using Euclidean distance, and the results visualized in a plot constructed from non-metric multidimensional scaling (nmMDS) analysis after checking stress and r^2 in a Shepard plot.

The DNA dataset was analyzed with the following variables: the number of species of each taxon, the total number of species, the proportion of exotic species over the total

number of species in each sample. Sequences assigned to terrestrial species, environmental DNA and assignment artifacts were excluded from the analysis. Comparisons of the average number of species on plastics (as plastic bags, plastic bottles, buoys and expanded polystyrene) and textile objects (including sanitary pads and fabric pieces) were done using non-parametric Mann-Whitney tests. The community inferred from metabarcoding was compared between pairs of items using Gower’s general similarity coefficient for presence-absence of each species, and nmMDS analysis was conducted as above. The same PAST software by Hammer et al. (2001) was employed.

Results

Beach litter

Beach surface ranged from 2500 m^2 in El Rinconin to 17500 m^2 in La Ñora. A total of 1023 litter objects were found in the beaches, corresponding to densities between 1.26 and 4.57 items/m^2 in Arbeyal and Peñarrubia respectively (Table 1). Considering the litter surface, it was between 2.46 cm^2 of litter per m^2 of beach in the cleanest Arbeyal to 18.6 in the most littered Peñarrubia (Table 1); for litter surface La Ñora joined the group of more polluted beaches together with Peñarrubia and Rinconín, while for the number of items it was closer to the least polluted Arbeyal and Cagonera.

Table 1. Characteristics of the beaches sampled from the central south Bay of Biscay. Beach surface in m^2. The litter density is given in surface as cm^2 of litter per m^2, and as litter items per m^2.

	**Arbeyal**	**Rinconín**	**Peñarrubia**	**Cagonera**	**La Ñora**
Type of beach	Urban	Urban	Rural	Rural	Rural
Substrate	Sand	Sand	Pebble	Pebble	Sand
River	No	No	No	No	Yes
Beach surface	14000	2500	8250	10625	17500
Latitude	43.5445N	43.5483N	43.5518N	43.5501N	43.5471N
Longitude	5.6934W	5.6390W	5.6237W	5.6100W	5.5897W
Litter density (surface)	2.46	9.01	18.61	2.93	10.46
Litter density (items)	1.26	4.20	4.57	1.29	1.30

The majority of litter was made of plastic (61.9%), 33.9% was textile (sanitary pads, clothes) and only 43 objects (4.2%) were of other materials. The five beaches were significantly different among each other for the type of litter (Chi-square = 837.94 with 40 degrees of freedom and P = $6.31x10^{-150}$; Monte Carlo P = 0.0001). For example, in Cagonera there were more textile items, while in La Ñora the predominant litter was small plastic pieces (Fig. 2A). Abandoned, lost or otherwise discarded fishing gears (plastic ALDFG) were found in all the beaches except in Arbeyal (the urban beach closer to Gijón port). Metallic objects, like cans, were scarce. They were found only from Rinconin beach (Fig. 2A). Biota composition was quite different among beaches, being Peñarrubia and Cagonera beaches the most similar ones, mainly containing species belonging to classes Florideophyceae and Dinophyceae (Fig. 2B).

Fig. 2 Litter (A) and biota (B) composition in the five beaches analyzed in this study, presented as proportion of each type of item (A) or proportion of species of each main taxonomic group (B).

The litter profile in the beach of Cagonera with so many sanitary pads can be explained from a punctual malfunctioning of the domestic wastewater treatment in the neighborhood. The neighbors were consulted about this and explained that the local wastewater treatment plant was temporarily closed and the toilets flushed directly to the beach. Thus, the large proportion of textile litter in that beach is likely not representative of the common beach state. Campaigns for not disposing this type of objects in toilets should be conducted in this area.

The nmMDS based on Euclidean distances had stress of 0, r^2 of 0.865 and 0.002 for the axis 1 and 2 respectively. The beaches were grouped roughly by the abundance of textile versus plastics, being connected in the minimum spanning tree almost following the relative proportion of textile (Fig. 3A); the beaches richer in plastics (Rinconín and La Ñora) were more or less proximate and separate of the former, and Peñarrubia was apart (Fig. 3A).

Fig. 3 Non-metric multidimensional scaling analysis of the litter composition (A) and the litter biofilm biota identified from DNA (B), in the five analyzed beaches. Scatter plots constructed from pairwise Gower distances. The minimum spanning tree is presented.

Biota on litter items identified from Next Generation Sequencing

The surfaces sampled for biofilm and their composition are presented in Table 1. In total they corresponded to 16 types from the different beaches, accounting for approximately 2.5‰ of the litter surface. Only biofilms from 12 samples (from the initial 16 samples) provided DNA of quality to be successfully PCR-amplified and sequenced (Table 2). Thus DNA sequences were not obtained from four expanded polystyrene pieces. For the 12 remaining biofilm samples, nine were from plastic objects and three were from textiles.

The initial screening left 278 124 sequences that were useful for species assignments, as they passed the quality filter being >200bp and with a quality value >20 (Table 2). Although the same DNA amount of each sample library was employed for next generation sequencing, results were dissimilar, as for some samples much more sequences were obtained than from others (Table 2). The polystyrene pieces from Peñarrubia (P-P3) was the sample for which more sequences were obtained (> 90000), while the plastic fragments from Cagonera (C-P4) provided the smallest number of sequences. After OTU assignation 66% of the sequences in P-P3 were lost (still remaining > 30000 sequences), and for the sample C-P4 none of the sequences assigned to a species with the BLAST criteria employed. So finally, biofilm communities were inferred from only 11 samples.

Table 2. Raw and filtered NGS results. Litter surfaces used for biofilm analyses, concentration of eDNA as ng/µL, number of reads obtained before and after quality filters, and number of sequences assigned taxonomically after the final BLAST.

Beach	Litter type	Material	Code	eDNA concentration (ng/µL)	Before quality filter	After quality filter	After BLAST (%97, <E^{-50})
La Ñora	Plastic Bottle	Plastic	Ñ-P1	2.66	57596	8476	969
	Plastic Bag	Plastic	Ñ-P2	1.45	110931	33004	3417
	Sanitary pad	Textile	Ñ-T1	2.09	8024	5915	97
	Expanded polystyrene	Plastic	Ñ-P3	-	-	-	-

Cagonera	Plastic fragment	Plastic	C-P4	1.88	1597	540	0
	Fishing gear	Plastic	C-P5	2.22	99716	38098	1379
	Sanitary pad	Textile	C-T1	1.98	93725	38877	3137
	Expanded polystyrene	Plastic	C-P3	-	-	-	-
Peñarrubia	Fabric piece	Textile	P-T2	1.79	14414	8501	1193
	Expanded polystyrene	Plastic	P-P3	5.63	370143	91354	30241
	Plastic Bottle	Plastic	P-P1	3.54	103369	24871	366
Rinconín	Buoy	Plastic	R-P5	2.09	88324	19754	53
	Expanded polystyrene	Plastic	R-P3	-	-	-	-
Arbeyal	Plastic Bag	Plastic	A-P2	2.17	5332	707	81
	Plastic fragment	Plastic	A-P4	3.45	21964	8027	131
	Expanded polystyrene	Plastic	A-P3	-	-	-	-

Species assignations made with a minimum identity of 90% and an E- value of $1e^{-10}$ retrieved many hits (S1 Table), but the reliability was too low because 82% of the manual individual BLAST did not assign the OTU to the same species. For > 97% identity with the same E- value of $1e^{-10}$, despite much fewer significant hits retrieved, still 45% of the sequences checked manually were assigned to a different species using manual BLAST. With a more stringent E-value of $1e^{-50}$ and 90% identity, the number of discrepancies between QIIME pipeline and the manual BLAST individual assignations was 22%. Finally, with an E-value $1e^{-50}$ and 95% identity all the putative species identified from QIIME coincided with those retrieved from manual BLAST. However, in order to increase the assignments to species level and not only to genus a minimum identity of 97% was chosen, being the final conditions for the metabarcoding assignments using QIIME a minimum identity of 97% and E-value of $1e^{-50}$. Although 85% of the initial sequences were lost due to these highly stringent parameters the identifications obtained were very robust, as deduced from total coincidence with the manual BLAST.

After all the process, 122 species were identified from biofilm DNA in the litter biofilms sampled. *Homo sapiens* and other non-marine species were detected, such as insects, mammals and freshwater organisms but they were not considered for posterior analyses (S2 Table). Since we were working with debris like sanitary pads or plastic bottles, which are in contact with humans, we expected to obtain a lot of human

sequences. Potential contamination with human DNA throughout the processing of samples can be discarded since no DNA amplification was detected in the negative control. Insect species (especially Diptera) and big mammals like *Bos taurus* (cattle) and *Sus scrofa* (wild boar) were found in rural beaches like Cagonera; likely runoffs carried the environmental DNA from the land. In the case of insects there is also the possibility that DNA belongs to eggs that adults laid on the debris.

Considering only marine and brackish taxa, 86 species classified into 17 major groups were identified from the biofilm samples analyzed. The putative taxa were not equally distributed in all the samples and beaches (Table 3). In fact, some items showed a bigger amount of taxa than others. Sanitary pads from Cagonera (C-T1) provided more species (44 species) than the rest. On the other hand, biofilm from plastic bags from Arbeyal (A-P2) only appeared to have a Phaeophycean alga (*Petalonia fascia*).

Table 3 Number of marine species of the main taxa obtained from each litter sample. The same species may appear several times in different samples. Litter sample codes as in Table 2.

	Sample										
**Taxon**	**A-P2**	**Ñ-P2**	**P-P1**	**C-T1**	**R-P5**	**Ñ-P1**	**C-P5**	**A-P4**	**P-T2**	**Ñ-T1**	**P-P3**
Polichaeta	0	0	0	1	0	0	0	0	1	0	1
Arthropoda	0	2	1	5	0	2	1	1	1	0	3
Apicomplexa	0	0	1	0	0	0	0	0	0	0	0
Ascomycota	0	2	1	4	0	0	0	0	0	0	1
Bacillariophyta	0	0	0	1	1	0	0	1	1	0	0
Bryozoa	0	0	0	1	0	0	0	1	2	2	0
Chordata	0	0	0	2	0	0	0	0	0	0	0
Cnidaria	0	0	0	4	1	0	0	0	0	0	1
Echinodermata	0	0	0	2	0	0	0	0	0	0	0
Mollusca	0	0	0	2	2	0	0	0	1	0	2
Dynophyceae	0	0	0	10	0	0	2	0	0	0	10
Florideophyceae	0	0	0	7	1	0	8	0	6	4	1
Amoebozoa	0	0	1	1	0	0	0	1	0	0	0
Nemertea	0	1	0	1	0	1	0	0	1	0	3
Phaeophyceae	1	0	0	0	0	0	0	2	1	0	1
Porifera	0	1	0	3	0	0	0	0	0	0	0
Bangiophyceae	0	0	0	0	1	0	0	0	0	0	0
**Total**	1	6	4	44	6	3	11	6	14	6	23

The non-metric scaling analysis arranged the beaches from their biofilm biota in an order similar to that found from the litter items (Figs. 3A and 3B), with La Ñora, Rinconin and Arbeyal connected closer than Cagonera and finally Peñarrubia. This was connected with different types of biota found in biofilm from textile and from plastic litter. For example, more Florideophycean (red algae) species were found on textile samples than on plastic ones (13 species were found on textile samples and only 9 on plastic; Figures 4A and 4B). For Dinophyceans, more species were found on plastic litter (Figure 4A) than in textiles (Fig. 4B). Only one species of Bangiophyceans appeared, which was found on plastic from Rinconin beach. On the other hand, the two species of echinoderms and the DNA of two species of Chordata (two Perciformes) that were found, only appeared on textile litter.

Fig. 4 Biota occurring in plastic (A, above) and textile (B, below) litter surfaces, determined from eDNA and NGS.

Textiles and plastics were compared for the number of species of each taxonomic group. Statistically significant differences between the two groups of litter items were found only for Bryozoans and Florideophyceae DNA from a significantly higher number of species of these taxa occurred on textile items than on plastic ones (Mann-Whitney $U = 0.5$ with $z = 2.764$, $P = 0.006$; and $U = 3$ with $z = 2.062$, $P = 0.03$, for Bryozoans and red algae respectively). However, focusing only on the macrofauna profiles analyzed in previous studies in the region (i.e. number of species of the phyla Annelida, Arthropoda, Bryozoa, Chordata, Cnidaria, Echinoderma, Mollusca, Porifera published in Miralles et al. 2016), they were not significantly different between textile and plastic (Chi square = 7.885, 6 d.f., $P = 0.247$, and Fisher's exact test with $P = 0.249 > 0.05$, not significant).

DNA of three non-native species was found in the dataset, including two species that are nowadays considered invasive in the study region: the brown alga *Sargassum muticum* and the signal crayfish *Pacifastacus leniusculus* (Table 4). The two marine alien species were found on Peñarrubia beach, attached to polystyrene items. The polystyrene containing DNA traces of *Illex argentinus* was a fragment of a box typically employed to transport fishing products and was not considered further, since the origin of DNA was likely from catch of seafood remains and not true South American squid larvae. On the other hand, DNA assigned to the signal crayfish (*Pacifastacus leniusculus*), which is

from brackish or fresh waters, was found on biofilm from plastic bottle in La Ñora beach near the river.

Table 4. Non-indigenous and nuisance species which DNA was found attached to beached litter objects; Shaded in gray, species native from the study region that have been described as aliens or invasive elsewhere.

Taxon	Species	Issue	Sample	Reference
Malacostraca	*Pacifastacus leniusculus*	Invasive	Ñ-Plastic Bottle	[48]
Cephalopoda	*Illex argentinus*	Alien	P- Polystyrene	[49]
Phaeophyceae	*Sargassum muticum*	Invasive	P- Polystyrene	[48]
Apicomplexa	*Isospora* sp.	Human parasite	P- Plastic Bottle	[50]
			Ñ-Plastic Bag	[51]
Ascomycota	*Cladosporium herbarum*	Asthmatic outbreaks and allergies	P- Polystyrene	[51]
			P- Polystyrene	[51]
Ascomycota	*Penicillium digitatum*	Rare pneumonia cases	C- Sanitary pad	[52]
Ascomycota	*Fusarium solani*	Infection of human cornea	C- Sanitary pad	[53]
Cnidaria	*Muggiaea atlantica*	Invasiveness reported in Germany	C- Sanitary pad	[54]
Bivalvia	*Mytilus edulis*	Alien in the Black Sea	C- Sanitary pad	[55]
Dynophyceae	*Alexandrium catenella*	Paralytic shellfish poisoning	P- Polystyrene	[56]
Dynophyceae	*Karenia brevis*	Respiratory irritation	C- Sanitary pad	[56]
Dynophyceae	*Peridinium* sp.	Toxic blooms	P- Polystyrene	[56]
Dynophyceae	*Alexandrium ostenfeldii*	Paralytic shellfish poisoning	C- Sanitary pad	[56]
Dynophyceae	*Karlodinium* sp.	Toxic blooms	C- Sanitary pad	[56]
Dynophyceae	*Alexandrium minutum*	Toxic PSP blooms	C- Sanitary pad	[56]
			C-Fishing Gear	[56]
Dynophyceae	*Alexandrium sp.*	May produce toxic blooms	P- Polystyrene	[56]
Dynophyceae	*Azadinium poporum*	Azaspiracid shellfish poisoning	C- Sanitary pad	[56]
Dynophyceae	*Prorocentrum micans*	Shellfish killing blooms	P- Polystyrene	[57]
Dynophyceae	*Scrippsiella sp.*	May produce high density blooms	P- Polystyrene	[58]
Dynophyceae	*Alexandrium affine*	Alien in China, Ukraine, California	C-Fishing Gear	[13]
Florideophyceae	*Plocamium cartilagineum*	Alien in the Mediterranean Sea	C- Sanitary pad	[13]
Florideophyceae	*Ellisolandia elongata*	Alien in the Belgian coast	R-Buoy	[59]
Florideophyceae	*Jania rubens*	Alien in the Mediterranean Sea	P-Fabric piece	[60]
Florideophyceae	*Chondrus crispus*	Alien in the United Kingdom	C-Fishing Gear	[61]
Florideophyceae	*Gymnogongrus crenulatus*	Alien in the Australian coast	C-Fishing Gear	[62]
Phaeophyceae	*Leathesia marina*	Alien in the Mediterranean Sea	A-Plastic fragment	[63]

Apart from these non-indigenous species, DNA from several native species was also found attached to the litter. Many of these species are considered potentially harmful because some strains can form toxic blooms (case of some dinoflagellate species), or produce diseases or allergies (Table 4). Some species do not cause any known toxicity

or nuisance effects, but they are considered potentially dangerous in different places around the world where they are non-indigenous or even invasive species. We detected Florideophycean species such as *Plocamium cartilagineum, Jania rubens* (aliens in the Mediterranean Sea), *Chondrus crispus* (alien in the United Kingdom) and *Gymnogongrus crenulatus* (alien in the Australian coast); molluscs (*Mytilus edulis*; alien in the Black Sea); and cnidarians (*Muggiaea atlantica*; invasion reports in Germany).

Litter as a vector for species dispersal from Gijon port

For exploring the possibility of marine litter being a vector of dispersal from the ports, the taxonomic profiles found in this study from beach litter were compared with published data from the port of Gijon. The comparison was done using the subset of marine macroscopic animal species only, because only macroscopic sessile animals were sampled using a conventional approach in Miralles et al. (2016). A total of 24 species were published in the port [39] (S3 Table). The number of shared species across taxonomic groups found in litter biofilm in the five beaches and in the port was four out of a total of 44 macroscopic animal species, corresponding to the polychaetes *Platynereis dumerilii* and *Syllis gracilis*, the mussel *Mytilus edulis*, and the limpet *Patella vulgata*. For further analysis, the macrofaunal species fouling on litter (whatever litter type, since no significant differences were found between textile and plastic for macrofauna species profiles) were organized by proximity to the port, considering together the beaches located in the same bay -Arbeyal, Rinconin and Peñarrubia on one group, Cagonera and La Ñora on the other (see Fig. 1). The profile of the fouled macroscopic fauna of the port and the litter found on closer beaches was more similar to each other than the fauna of the litter found on farther beaches (Fig. 5). The macrofauna profile of Gijon port published by Miralles et al. (2016) was not significantly different of that found on litter from the three closer beaches (Chi square = 7.797, 5 d.f., P = 0.168, and Fisher's exact test with P = 0.193). In contrast, the taxonomic profile of the litter macrofauna found from the farther La Ñora and Cagonera beaches was highly significantly different from the port fauna reported by Miralles et al. (2016) (Chi square = 27.051, 7 d.f., P = 0.0003, and Fisher's exact test with P = 1.91×10^{-5}).

Fig. 5 Proportion of species of different animal groups fouling Gijon old port piers and litter from beaches near (Litter - close) or apart (Litter - far) the port.

Discussion

Although based on a modest number of items, this study provided a number of results of importance in the field of environmental security. In the biofilm communities, as expected, some of the species detected from DNA are microscopic, such as amoebas or dinophytes. For the macroscopic species, the DNA was probably provided from free environmental DNA of macroscopic organisms, or microscopic phases (early larvae, eggs, propagules). The first result to be highlighted was DNA of a significantly higher number of red algae and bryozoan species found in textile debris than in plastic litter. Taking into account that both red algae [64-65], and bryozoans [66-67] contain a high proportion of invasive species, it seems that textile debris would have the potential to be a reservoir of potentially invasive species. Moreover, some of the species found in textile are dangerous for public health because they may cause red tides (e.g. *Alexandrium minutum*) or produce infections (e.g. *Fusarium solani*, *Cladosporium herbarum*), thus the role of textile litter as a reservoir of species should be carefully taken into account. Although fabric floatability is in principle lower than that of plastics, thus a priori having shorter dispersal capacity, in beaches with high litter accumulation the species accumulated in textile may pass on plastic items and eventually navigate offshore, live tides or storms occurring. Nowadays focused on massive plastic debris, future studies should consider also other types of litter –in addition to plastics- in order to fully understand the role of marine litter as reservoir and dispersal vector of nuisance species.

Another interesting result was the higher similarity of litter macrofauna profile with port's macrofauna in closer beaches than in farther ones. This can be considered a signal of species dispersal from the port using marine litter as vectors. The macrofauna species found on litter from their DNA were all native or cosmopolitan, this suggesting that litter could not only transport alien species from Bay of Biscay ports [36], but also serve as a vector for the dispersal of native species, as it was found in Swedish waters [68].

Moreover, some of the native species that were found attached to marine litter (mainly Florideophyceans) are considered alien or invasive in many other zones around the globe, so our results show that marine litter could be potentially dangerous in these areas, as it could be used as a spreading vector, facilitating alien species to reach and colonize new habitats.

Regarding local alien species, in the NGS results we detected DNA of several non-indigenous species, including an alga (*Sargassum muticum*), a cephalopod (*Illex argentinus*), and a freshwater crayfish (*Pacifastacus leniusculus*). Alien species tend to be very difficult to identify in the initial phases of colonization, as their population size is normally small. This is an important issue, because nonindigenous species eradication is easier in the first introduction stages when the population is not too big [69]. Sequences as those found in this study that occurred with low frequency should be taken into account, as they might be the key to anticipate or avoid possible future invasions. In this sense a deeper analysis is needed to interpret correctly the presence of DNA of exotic species on the particular litter objects analyzed in this study. The polystyrene piece sampled in Peñarrubia carried 39 DNA sequences of two alien species identified as *Illex argentinus* and *Sargassum muticum.* Individual BLASTs were made with some of the sequences belonging to *Illex argentinus* and confirmed that they were all correctly assigned. However, the Argentinean squid has no sessile larvae, and the species has never been detected in the Bay of Biscay. The origin of the polystyrene could explain this result; this material is employed in fishing vessels - polystyrene boxes are used to store the catch. Probably a polystyrene box used to store that squid ended on the sea and arrived in Peñarrubia beach, still containing rests of squid DNA in the biofilm.

In contrast with the former aliens, the other two are considered invasive in Spanish waters. *Sargassum muticum* is a brown seaweed that has been already detected in Asturias [70] and alters local biodiversity triggering the decline of some native species such as *Gelidium spinosum* [71]. Our results suggest small propagules of this species could be transported attached to marine litter, using it as a spreading vector to colonize new environments. On the other hand, the presence of DNA of the freshwater signal crayfish in a plastic bottle (household origin) from La Ñora beach can be easily explained. This beach is in the estuary of River La Ñora, and eggs, larvae or naked

DNA from freshwater organisms can arrive from the river, as rivers are conveyor belts of DNA diversity [72]. The species *Pacifastacus leniusculus* has been reported from River La Ñora [73] and our results are consistent with it, having a representation of the species living upstream.

On the technical side, next-generation sequencing was carried out from miniCOI amplicons in this study. COI is a largely studied gene, and big amount of sequences are available [74]. Reference databases for the 18S gene are currently growing and the gene has been incorporated for example in BOLD (Barcoding of Life Diversity) although more recently than COI, thus the number of reference sequences is still smaller for 18S gene [75]. For this reason, we based our study only on COI, that is today the most represented DNA barcode in public databases [76]. Although COI gene is not a marker of choice for algae identification, due to its low variability among plant species [77], in this work we obtained many algae sequences with a high reliability (minimum identity higher than 95% and e value $<10^{-50}$). Many of them were randomly reviewed with individual BLAST and gave a robust assignment with >97% identity and high scores, so our study aligns with other authors who found COI to be a good tool to sequence red algae such as Florideophyceae that we obtained here [78].

A problem for the use of Metabarcoding in biodiversity inventories is the unbalanced coverage of different taxonomic groups in current reference databases, especially in aquatic species [76]. Three biofilm sequences of a sanitary pad were assigned to *Squamamoeba japonica* (S1 Table). Its presence in coastal Bay of Biscay waters could be difficult to explain, because it is a deep-sea Pacific amoeba [79]. This could be an assignment artifact due to the scarcity of references because in July 2019 the only sea amoebas represented in GenBank with COI gene were of this species. It is possible that some DNA sequences of other marine amoebas were erroneously assigned to it.

A limitation of this study is that we cannot be sure if the individuals detected from DNA are alive or dead, that is, are or not able to start a real colonization of the surroundings of the litter items where they were found. DNA can persist extracellularly in the environment, making discrimination of living versus dead organisms difficult. In contrast, RNA molecules have a low durability in the environment, so a metabarcoding

approach using eDNA and eRNA may be used to differentiate between dead and alive organisms [80]. Further studies could use RNA instead of DNA molecules for species detection from marine litter.

Conclusions and management recommendations

In this study, species potentially dangerous for ecosystem and human health have been found from DNA analysis of biofilm fouling litter objects. Textile objects, although likely less mobile than plastic ones, carried a significantly higher proportion of nuisance species. On the other hand, the macrofauna profile of litter objects found on beaches seemed to be associated to the distance from the port, the closer the more similar. From these results, paying attention to textile litter objects would be recommended, in addition to the current concern about plastics and microplastics. Preventing litter dispersal from ports would be another important recommendation for avoiding exotic species spread.

References

1. Pimm, S. L., Russell, G. J., Gittleman, J. L., & Brooks, T. M. (1995). The future of biodiversity. *Science, 269*(5222), 347.
2. Moser, M. L., & Lee, D. S. (1992). A fourteen-year survey of plastic ingestion by western North Atlantic seabirds. *Colonial Waterbirds,* 83-94.
3. Bugoni, L., Krause, L., & Petry, M. V. (2001). Marine debris and human impacts on sea turtles in southern Brazil. *Marine Pollution Bulletin, 42*(12), 1330-1334.
4. Mattlin, R. H., & Cawthorn, M. W. (1986). Marine debris—an international problem. *New Zealand Environment*, *51*, 3-6.
5. Fowler, C. W. (1987). Marine debris and northern fur seals: a case study. *Marine Pollution Bulletin*, *18*(6), 326-335.
6. Carr, A. (1987). Impact of nondegradable marine debris on the ecology and survival outlook of sea turtles. *Marine Pollution Bulletin*, *18*(6), 352-356.
7. Mouat, J., Lozano, R. L., & Bateson, H. (2010). *Economic impacts of marine litter*. Kommunenes Internasjonale Miljøorganisasjon.

8. Jang, Y. C., Hong, S., Lee, J., Lee, M. J., & Shim, W. J. (2014). Estimation of lost tourism revenue in Geoje Island from the 2011 marine debris pollution event in South Korea. *Marine pollution bulletin, 81*(1), 49-54.
9. Rech, S., Borrell, Y., & García-Vazquez, E. (2016). Marine litter as a vector for non- native species: What we need to know. *Marine Pollution Bulletin*, *113*(1), 40-43.
10. Barnes, D. K. (2002). Biodiversity: invasions by marine life on plastic debris. *Nature*, *416*(6883), 808-809.
11. Grassle, J. F., Lasserre, P., McIntyre, A. D., & Ray, G. C. (1991). Marine biodiversity and ecosystem function. Biology International Special, (23), 1-19.
12. Sakai, A. K., Allendorf, F. W., Holt, J. S., Lodge, D. M., Molofsky, J., With, K. A. et al. (2001). The population biology of invasive species. *Annual review of ecology and systematics*, *32*(1), 305-332.
13. Molnar, J. L., Gamboa, R. L., Revenga, C., & Spalding, M. D. (2008). Assessing the global threat of invasive species to marine biodiversity. *Frontiers in Ecology and the Environment*, *6*(9), 485-492.
14. Pimentel, D., Lach, L., Zuniga, R., & Morrison, D. (2000). Environmental and economic costs of nonindigenous species in the United States. *BioScience*, *50*(1), 53-65.
15. Verlaque, M. (1994). Checklist of introduced plants in the Mediterranean: Origins and impact on the environment and human activities. Oceanologica acta. Paris, 17(1), 1-23.
16. Walton, W. C., MacKinnon, C., Rodriguez, L. F., Proctor, C., & Ruiz, G. M. (2002). Effect of an invasive crab upon a marine fishery: green crab, Carcinus maenas, predation upon a venerid clam, Katelysia scalarina, in Tasmania (Australia). Journal of Experimental Marine Biology and Ecology, 272(2), 171-189.
17. Harvey, C. T., Qureshi, S. A., & MacIsaac, H. J. (2009). Detection of a colonizing, aquatic, non-indigenous species. Diversity and Distributions, 15(3), 429-437.
18. Jerde, C. L., Mahon, A. R., Chadderton, W. L., & Lodge, D. M. (2011). "Sight-unseen" detection of rare aquatic species using environmental DNA. *Conservation Letters*, 4(2), 150-157.

19. MacKenzie, D. I. (2006). *Occupancy estimation and modeling: inferring patterns and dynamics of species occurrence*. Academic Press.
20. Dejean, T., Valentini, A., Miquel, C., Taberlet, P., Bellemain, E., & Miaud, C. (2012). Improved detection of an alien invasive species through environmental DNA barcoding: the example of the American bullfrog Lithobates catesbeianus. *Journal of applied ecology*, *49*(4), 953-959.
21. Darling, J. A., & Blum, M. J. (2007). DNA-based methods for monitoring invasive species: a review and prospectus. *Biological Invasions*, *9*(7), 751-765.
22. Sogin, M. L., Morrison, H. G., Huber, J. A., Welch, D. M., Huse, S. M., Neal, P. R. et al. (2006). Microbial diversity in the deep sea and the underexplored “rare biosphere”. Proceedings of the National Academy of Sciences, 103(32), 12115-12120.
23. Andersson, A. F., Lindberg, M., Jakobsson, H., Bäckhed, F., Nyrén, P., & Engstrand, L. (2008). Comparative analysis of human gut microbiota by barcoded pyrosequencing. *PloS one*, *3*(7), e2836.
24. Hajibabaei, M., Shokralla, S., Zhou, X., Singer, G. A., & Baird, D. J. (2011). Environmental barcoding: a next-generation sequencing approach for biomonitoring applications using river benthos. *PLoS one*, *6*(4), e17497.
25. Somervuo, P., Yu, D. W., Xu, C. C., Ji, Y., Hultman, J., Wirta, H., & Ovaskainen, O. (2017). Quantifying uncertainty of taxonomic placement in DNA barcoding and metabarcoding. *Methods in Ecology and Evolution*, 8(4), 398-407.
26. Hering, D., Borja, A., Jones, J. I., Pont, D., Boets, P., Bouchez, A. et al. (2018). Implementation options for DNA-based identification into ecological status assessment under the European Water Framework Directive. *Water research.*
27. Hulme, P. E. (2006). Beyond control: wider implications for the management of biological invasions. Journal of Applied Ecology, 43(5), 835-847.
28. Ficetola, G. F., Miaud, C., Pompanon, F., & Taberlet, P. (2008). Species detection using environmental DNA from water samples. *Biology letters*, *4*(4), 423-425.
29. Carlton, J. T. (1996). Pattern, process, and prediction in marine invasion ecology. *Biological conservation*, *78*(1), 97-106.

30. Wonham, M. J., Walton, W. C., Ruiz, G. M., Frese, A. M., & Galil, B. S. (2001). Going to the source: role of the invasion pathway in determining potential invaders. *Marine Ecology Progress Series, 215, 1-12.*
31. Bravo, M., Astudillo, J.C., Lancellotti, D., Luna-Jorquera, G., Valdivia, N., Thiel, M., (2011). Rafting on abiotic substrata: Properties of floating items and their influence on community succession. *Mar. Ecol. Prog. Ser. 439, 1–17.*
32. Carson, H.S., Nerheim, M.S., Carroll, K.A., Eriksen, M., 2013. The plastic-associated microorganisms of the North Pacific Gyre. *Marine Pollution Bulletin. 75, 126–32.*
33. Zettler, E.R., Mincer, T.J., Amaral-Zettler, L.A., 2013. Life in the "plastisphere": Microbial communities on plastic marine debris. *Environ. Sci. Technol. 47, 7137–7146*
34. Reisser J, Shaw J, Hallegraeff G, Proietti M, Barnes DKA, et al. (2014) Millimeter- Sized Marine Plastics: A New Pelagic Habitat for Microorganisms and Invertebrates. PLoS ONE 9(6): e100289. doi:10.1371/journal.pone.0100289
35. Kiessling, T., Gutow, L., Thiel, M. (2015). Marine litter as habitat and dispersal vector, in: In: Bergmann M., Gutow L., Klages M. (eds) *Marine Anthropogenic Litter. Springer, Cham. pp. 141–180.*
36. Miralles L., Gomez-Agenjo M, Rayon-Viña F, Gyraite G, Garcia-Vazquez E. (2018) Alert calling in port areas: marine litter as possible secondary dispersal vector for hitchhiking invasive species. Journal for Nature Conservation 42: 12-18
37. Rech S, Borrell YJ, Garcia-Vazquez E. (2018) Anthropogenic marine litter composition in coastal areas may be a predictor of potentially invasive rafting fauna. PLoS ONE 13(1): e0191859.
38. Winston, J. E., Gregory, M. R., & Stevens, L. M. (1997). Encrusters, epibionts, and other biota associated with pelagic plastics: a review of biogeographical, environmental, and conservation issues. In *Marine Debris (pp. 81-97). Springer New York.*
39. Miralles, L., Ardura, A., Arias, A., Borrell, Y. J., Clusa, L., Dopico, E., ... & Valiente, A. G. (2016). Barcodes of marine invertebrates from north Iberian ports: Native diversity and resistance to biological invasions. *Marine pollution bulletin*, 112(1-2), 183-188.

40. Botas JA, Fernandez E, Bode A, Anadon R (1989). Water masses off the Central Cantabrian Coast. *Scientia Marina*, 53(4), 755-761.
41. Appeltans W, Bouchet P, Boxshall GA, Fauchald K, Gordon DP, Hoeksema BW, Poore GC, Van Soest RW, Stöhr S, Walter TC, Costello MJ. (2012). World register of marine species. *Accessed online: http://www. marinespecies. org (accessed on 24 May 2017).*
42. Nehring, S. (2006). NOBANIS–Invasive alien species fact sheet. *From: Online Database of the North European and Baltic Network on Invasive Alien Species-NOBANIS www. nobanis. org, (accessed on 24 May 2017).*
43. Pochon, X., Zaiko, A., Hopkins, G. A., Banks, J. C., & Wood, S. A. (2015). Early detection of eukaryotic communities from marine biofilm using high-throughput sequencing: an assessment of different sampling devices. Biofouling, 31(3), 241-251.
44. Leray, M., Yang, J. Y., Meyer, C. P., Mills, S. C., Agudelo, N., Ranwez, V., ... & Machida, R. J. (2013). A new versatile primer set targeting a short fragment of the mitochondrial COI region for metabarcoding metazoan diversity: application for characterizing coral reef fish gut contents. *Frontiers in zoology*, *10*(1), 34.
45. Caporaso JG, Kuczynski J, Stombaugh J, Bittinger K, Bushman FD, Costello EK, Fierer N, Pena AG, Goodrich JK, Gordon JI, Huttley GA. (2010). QIIME allows analysis of high-throughput community sequencing data. *Nature methods*, *7*(5), 335.
46. Hebert, P. D., Ratnasingham, S., & De Waard, J. R. (2003). Barcoding animal life: cytochrome c oxidase subunit 1 divergences among closely related species. Proceedings of the Royal Society of London. Series B: *Biological Sciences*, 270(suppl_1), S96-S99.
47. Hammer, Ø., Harper, D. A. T., & Ryan, P. D. (2001). PAST: Paleontological Statistics Software Package for Education and Data Analysis.[Computer program] Palaeontología Electrónica. Accessed online: http://palaeoelectronica. org/2001_1/past/issue1_01. htm (accessed on 26 May 2017).
48. Autoridad Portuaria de Gijón. (2017). El Puerto de Gijón - Autoridad Portuaria de Gijón. [online] Available at: https://www.puertogijon.es/puerto/ [Accessed 25 May 2018].

49. Van der Land, J. (ed). (2008). UNESCO-IOC Register of Marine Organisms (URMO).

50. Lindsay, D. S., Dubey, J. P., & Blagburn, B. L. (1997). Biology of Isospora spp. from humans, nonhuman primates, and domestic animals. *Clinical Microbiology Reviews*, 10(1), 19-34.

51. Breitenbach, M., & Simon-Nobbe, B. (2002). The allergens of Cladosporium herbarum and Alternaria alternata. In *Fungal allergy and pathogenicity* (Vol. 81, pp. 48-72). Karger Publishers.

52. Oshikata, C., Tsurikisawa, N., Saito, A., Watanabe, M., Kamata, Y., Tanaka, M. et al. (2013). Fatal pneumonia caused by Penicillium digitatum: a case report. *BMC pulmonary medicine*, 13(1), 16.

53. Howard, D. H. (Ed.). (2002). *Pathogenic fungi in humans and animals*. CRC Press.

54. Greve, W. (1994). The 1989 german bight invasion of muggiaea atlantica. *ICES Journal of Marine Science*, 51(4), 355-358.

55. Alexandrov, B., & Berlinsky, N. (2004). Basic biological investigations of Odessa maritime port (August-December, 2001): final report. *GloBallast Monograph Series*, (7).

56. Moestrup, Ø.; Akselmann, R.; Fraga, S.; Hoppenrath, M.; Iwataki, M.; Komárek, J.; Larsen, J.; Lundholm, N.; Zingone, A. (Eds) (2009 onwards). IOC-UNESCO Taxonomic Reference List of Harmful Micro Algae. Accessed on 2018-12-13 at http://www.marinespecies.org/hab .

57. Faust, M. A., & Gulledge, R. A. (2002). Identifying harmful marine dinoflagellates. *Contributions from the United States national herbarium*, 42.

58. Gárate-Lizárraga, I., Band-Schmidt, C. J., López-Cortés, D. J., & Muñetón-Gómez, M. D. S. (2009). Bloom of Scrippsiella trochoidea (Gonyaulacaceae) in a shrimp pond in the southwestern Gulf of California, Mexico. *Marine pollution bulletin*, 58(1), 145-149.

59. GRIIS. (2016). The Global Register of Introduced and Invasive Species. IUCN SSC Invasive Species Specialist Group.

60. Verlaque, M. (2001). Checklist of the macroalgae of Thau Lagoon (Hérault, France), a hot spot of marine species introduction in Europe. *Oceanologica Acta*. 24(1): 29-49.

61. Guiry, M. D. (2014). The seaweed site: information on marine algae. *Seaweed. ie*.
62. Sliwa, C.; Migus, S.; McEnnulty, F.; Hayes, K. R. (2009). Marine Bioinvasions in Australia. *Biological Invasions in Marine Ecosystems*. pp. 425-437.
63. Boudouresque, C. F.; Verlaque, M. (2002). Biological pollution in the Mediterranean Sea: invasive versus introduced macrophytes. *Marine Pollution Bulletin*. 44(1): 32-38.
64. Nyberg, C. D., & Wallentinus, I. (2005). Can species traits be used to predict marine macroalgal introductions? *Biological Invasions*, *7*(2), 265-279.
65. Williams, S. L., & Smith, J. E. (2007). A global review of the distribution, taxonomy, and impacts of introduced seaweeds. Annu. Rev. Ecol. Evol. Syst., 38, 327-359.
66. Watts, P. C., Thorpe, J. P., & Taylor, P. D. (1998). Natural and anthropogenic dispersal mechanisms in the marine environment: a study using cheilostome Bryozoa. Philosophical Transactions of the Royal Society of London B: Biological Sciences, 353(1367), 453-464.
67. Dyrynda, P. E. J., Fairall, V. R., Occhipinti Ambrogi, A., & d'Hondt, J. L. (2000). The distribution, origins and taxonomy of Tricellaria inopinata d'Hondt and Occhipinti Ambrogi, 1985, an invasive bryozoan new to the Atlantic. *Journal of Natural History, 34*(10), 1993-2006.
68. Garcia-Vazquez E, Cani A, Diem A, Ferreira C, Geldhof R, Marquez L, Molloy E, Perché S. (2018) Leave no traces – Beached marine litter shelters both invasive and native species. *Marine Pollution Bulletin* 131 Part A: 314-322.
69. Bax, N., Carlton, J. T., Mathews-Amos, A., Haedrich, R. L., Howarth, F. G., Purcell, J. E., ... & Gray, A. (2001). The control of biological invasions in the world's oceans. *Conservation Biology*, 15(5), 1234-1246.
70. Fernández, C., Gutiérrez, L. M., & Rico, J. M. (1990). Ecology of Sargassum muticum on the north coast of Spain. Preliminary observations. *Botanica marina*, *33*(5), 423-428.
71. Sanchez, I., Fernández, C., & Arrontes, J. (2005). Long term changes in the structure of intertidal assemblages after invasion by *Sargassum muticum* (Phaeophyta) 1. *Journal of Phycology, 41*(5), 942-949.

72. Deiner, K., Fronhofer, E. A., Mächler, E., Walser, J. C., & Altermatt, F. (2016). Environmental DNA reveals that rivers are conveyer belts of biodiversity information. *Nature communications*, 7, 12544.
73. Ayuntamiento de Gijón. (2016). Sendas verdes por el concejo de Gijón. [online] Available at: https://www.gijon.es [Accessed 28 May 2018].
74. Ratnasingham, S., & Hebert, P. D. (2007). BOLD: The Barcode of Life Data System (http://www. barcodinglife. org). Molecular Ecology Resources, 7(3), 355-364.
75. Bucklin, A., Steinke, D., & Blanco-Bercial, L. (2011). DNA barcoding of marine metazoa. *Annual Review of Marine Science, 3,* 471-508.
76. Weigand A., Beermann A.J., Čiampor F., Costa F.O., Csabai Z., Duarte S., et al. (2019) DNA barcode reference libraries for the monitoring of aquatic biota in Europe: Gap-analysis and recommendations for future work. Science of The Total Environment 678, 499-524.
77. Robba, L., Russell, S. J., Barker, G. L., & Brodie, J. (2006). Assessing the use of the mitochondrial cox1 marker for use in DNA barcoding of red algae (Rhodophyta). *American journal of botany, 93*(8), 1101-1108.
78. Saunders, G. W. (2005). Applying DNA barcoding to red macroalgae: a preliminary appraisal holds promise for future applications. *Philosophical transactions of the Royal Society B: Biological sciences*, *360*(1462), 1879-1888.
79. Kudryavtsev, A.; Pawlowski J. (2013). Squamamoeba japonica (Amoebozoa): a deep-sea amoeba from the Sea of Japan with a novel cell coat structure. *Protist,* 164(1), 13-23.
80. Pochon, X., Zaiko, A., Fletcher, L. M., Laroche, O., & Wood, S. A. (2017). Wanted dead or alive? Using metabarcoding of environmental DNA and RNA to distinguish living assemblages for biosecurity applications. *PloS one*, 12(11), e0187636.

Supporting information

S1 Table. Number of sequences assigned to a species level with different BLAST criteria. Beach acronyms stand as A, C, Ñ, P and R for Arbeyal, Cagonera, Ñora, Peñarrubia and Rinconin respectively.

S2 Table. Number of sequences assigned to each species in the biofilm samples analyzed.

S3 Table. Macroscopic animal species found from conventional sampling in Gijón port (Miralles et al. 2016) and from eDNA and NGS on litter biofilms in Gijón beaches.

Figure 1

Click here to download Figure Figure 1.tiff

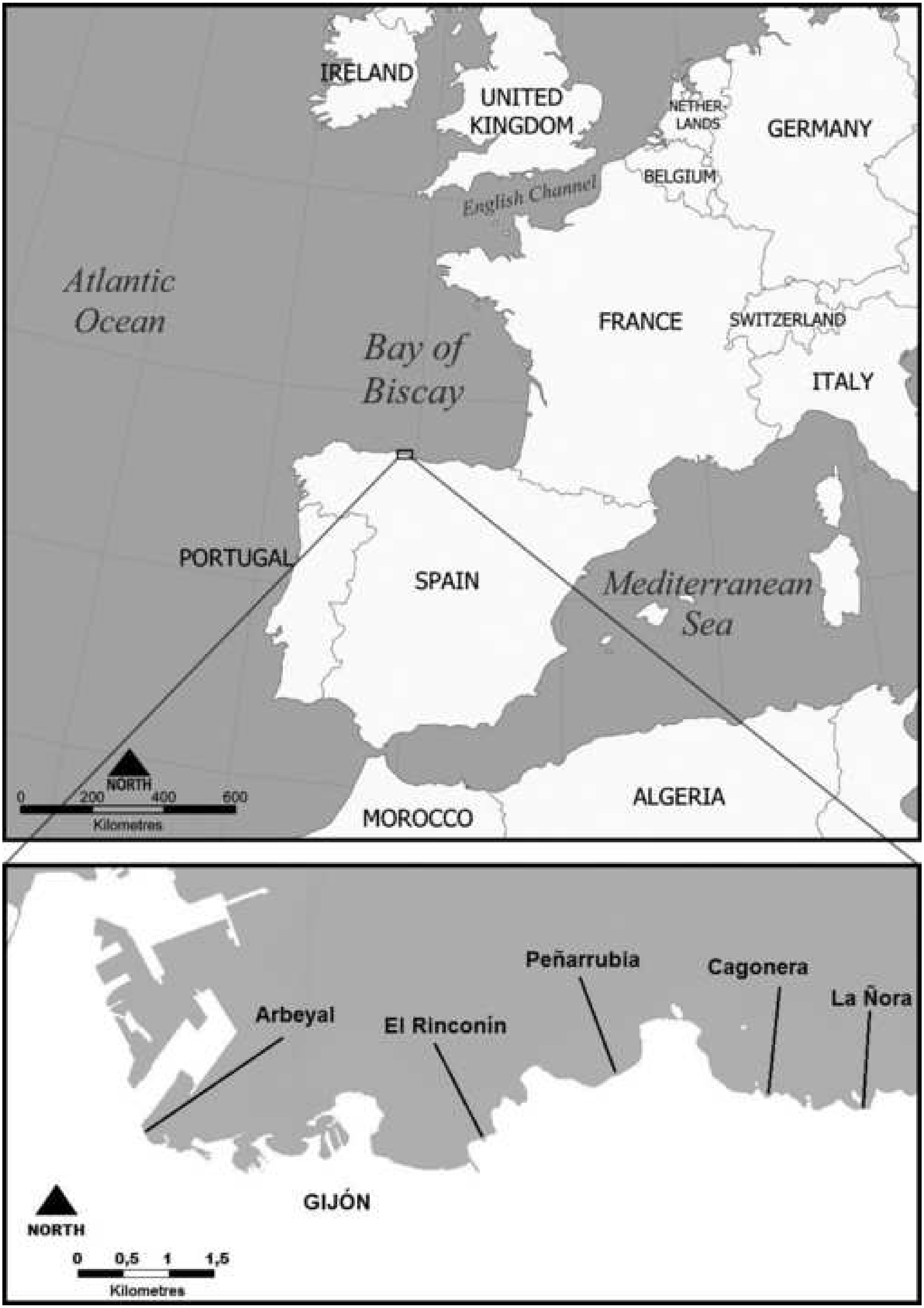

Figure 2A

Click here to download Figure Figure 2A.tiff

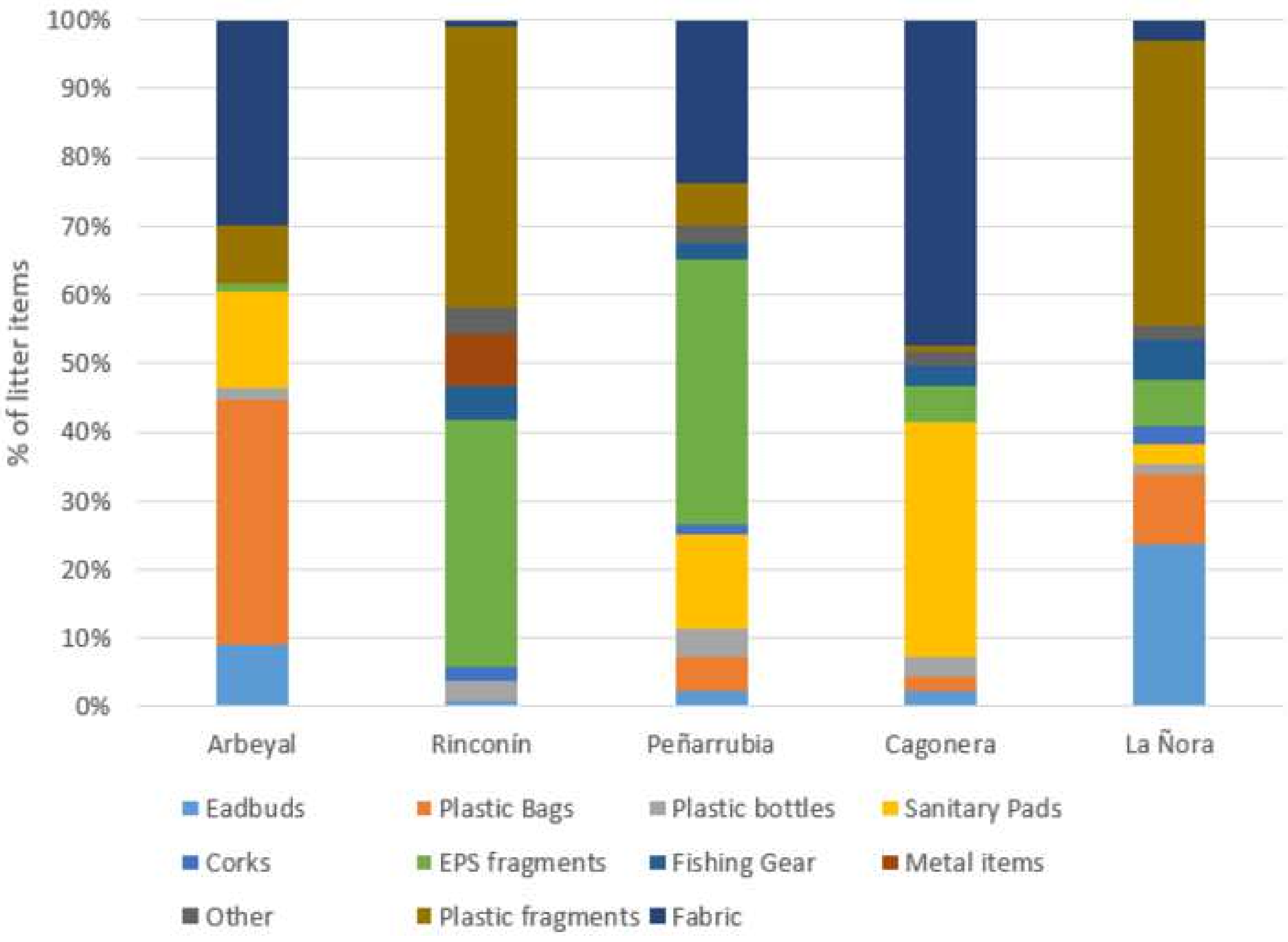

Figure 2B

Click here to download Figure Figure 2B.tiff

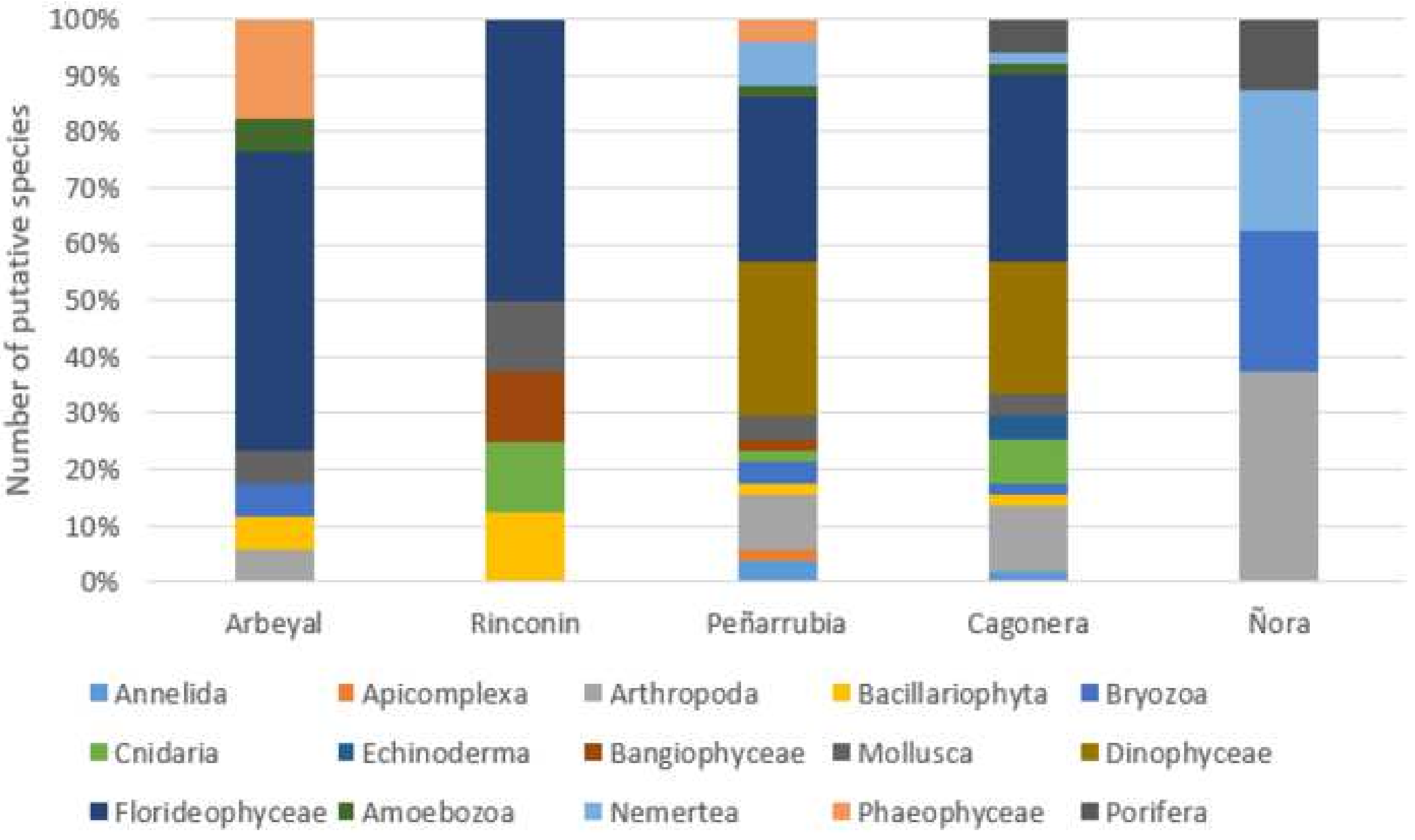

Figure 3A

Click here to download Figure Figure 3A.tiff

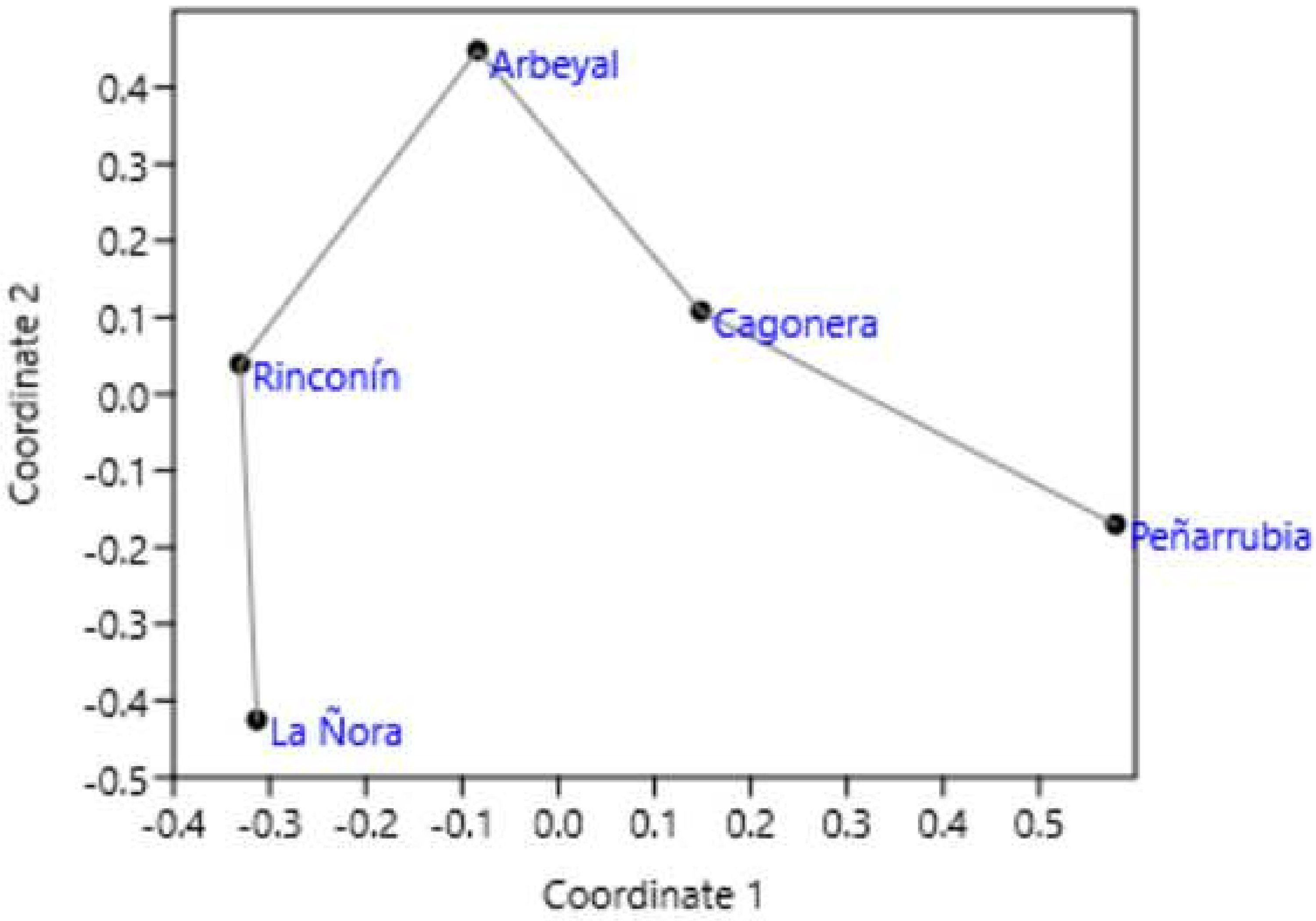

Figure 3B

Click here to download Figure Figure 3B.tiff

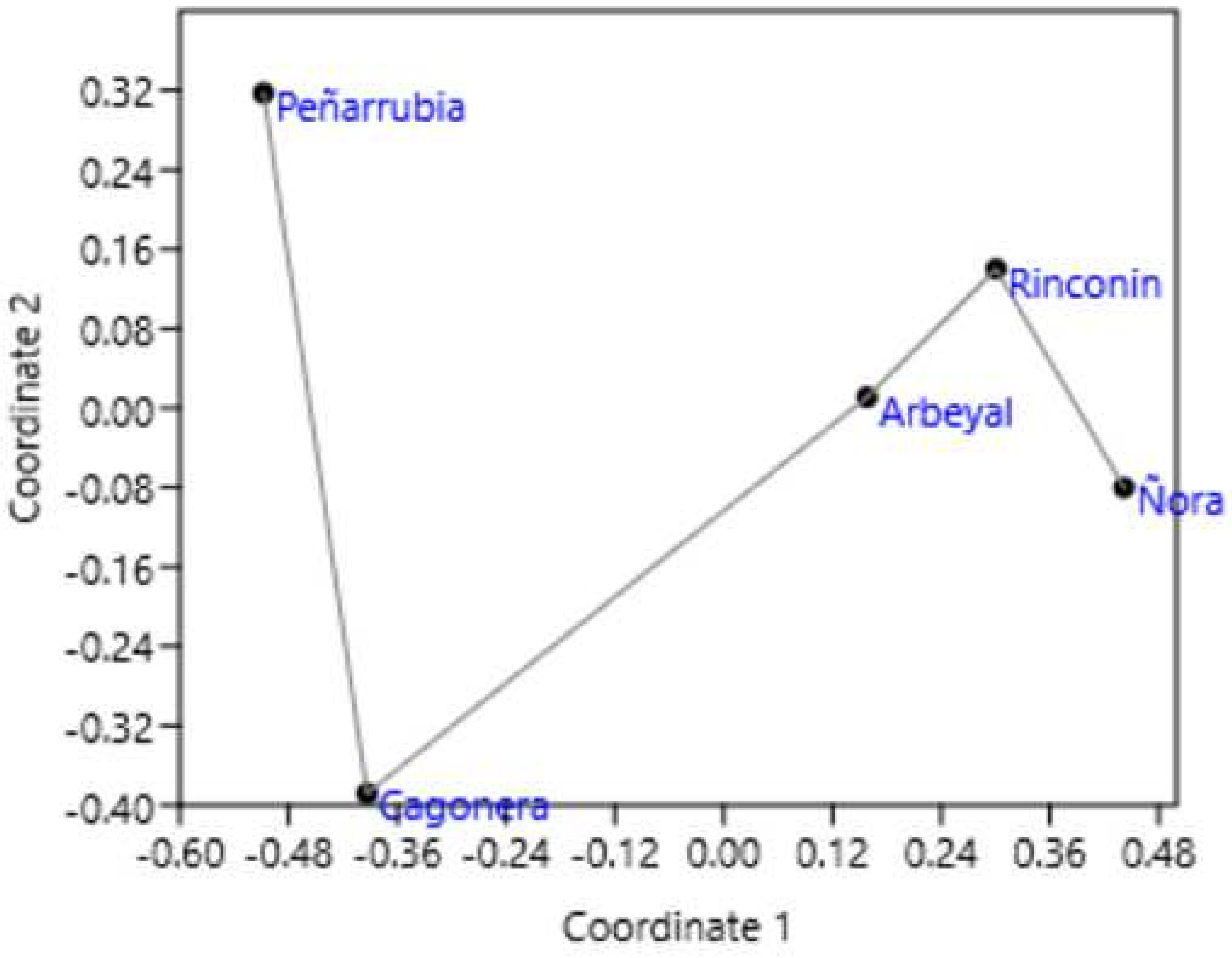

Figure 4A

Click here to download Figure Figure 4A.tiff

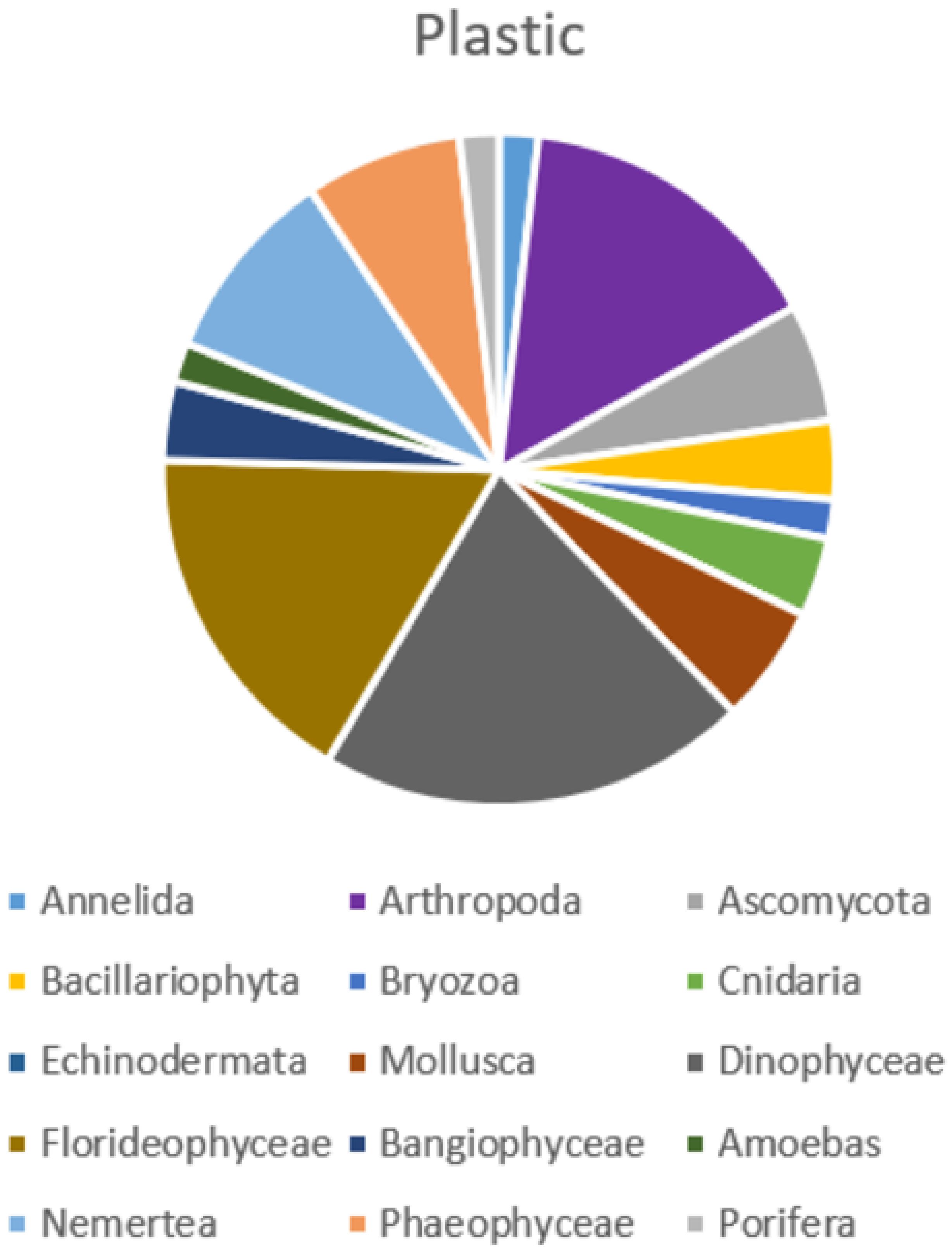

Figure 4B

Click here to download Figure Figure 4B.tiff

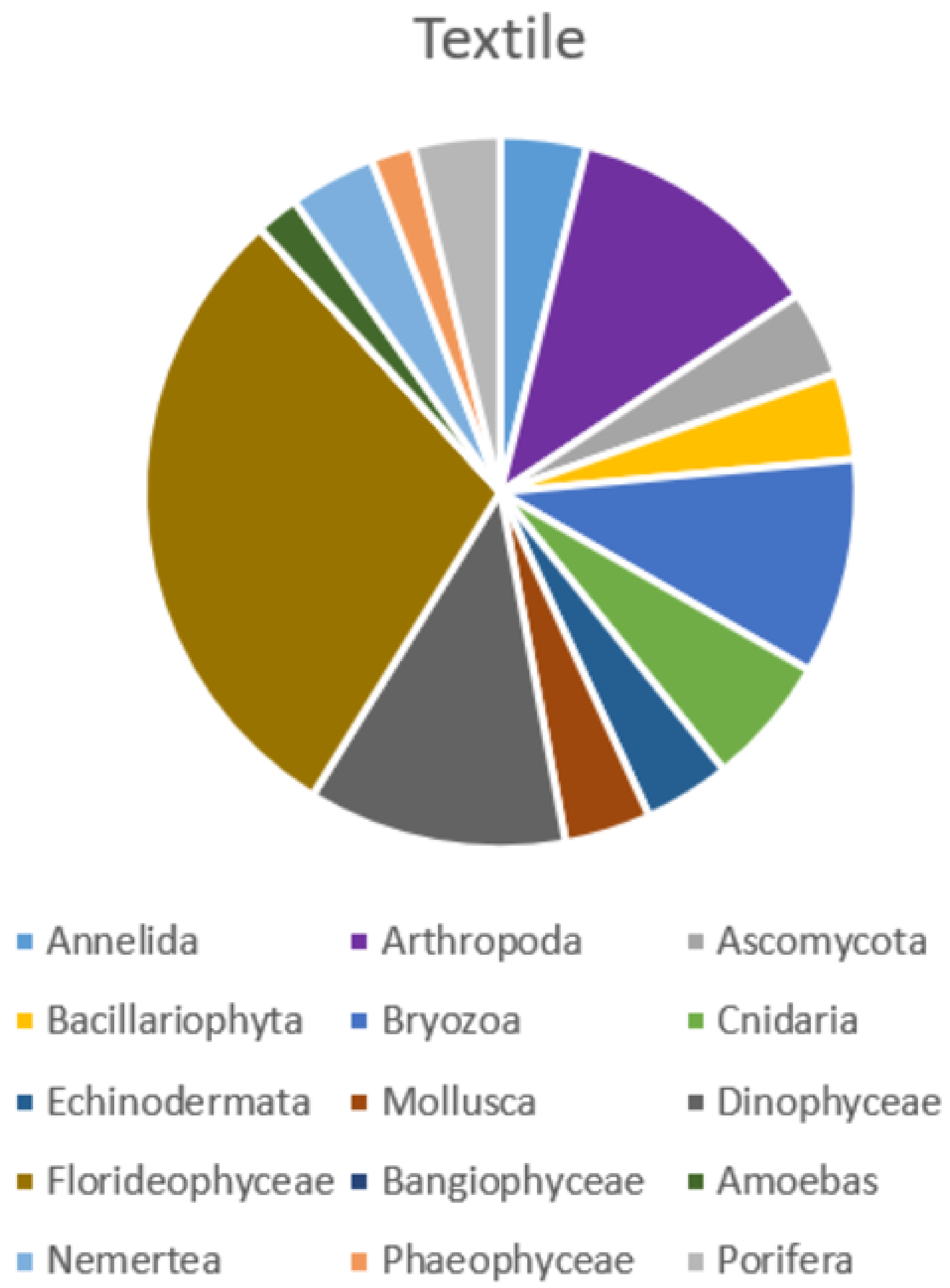

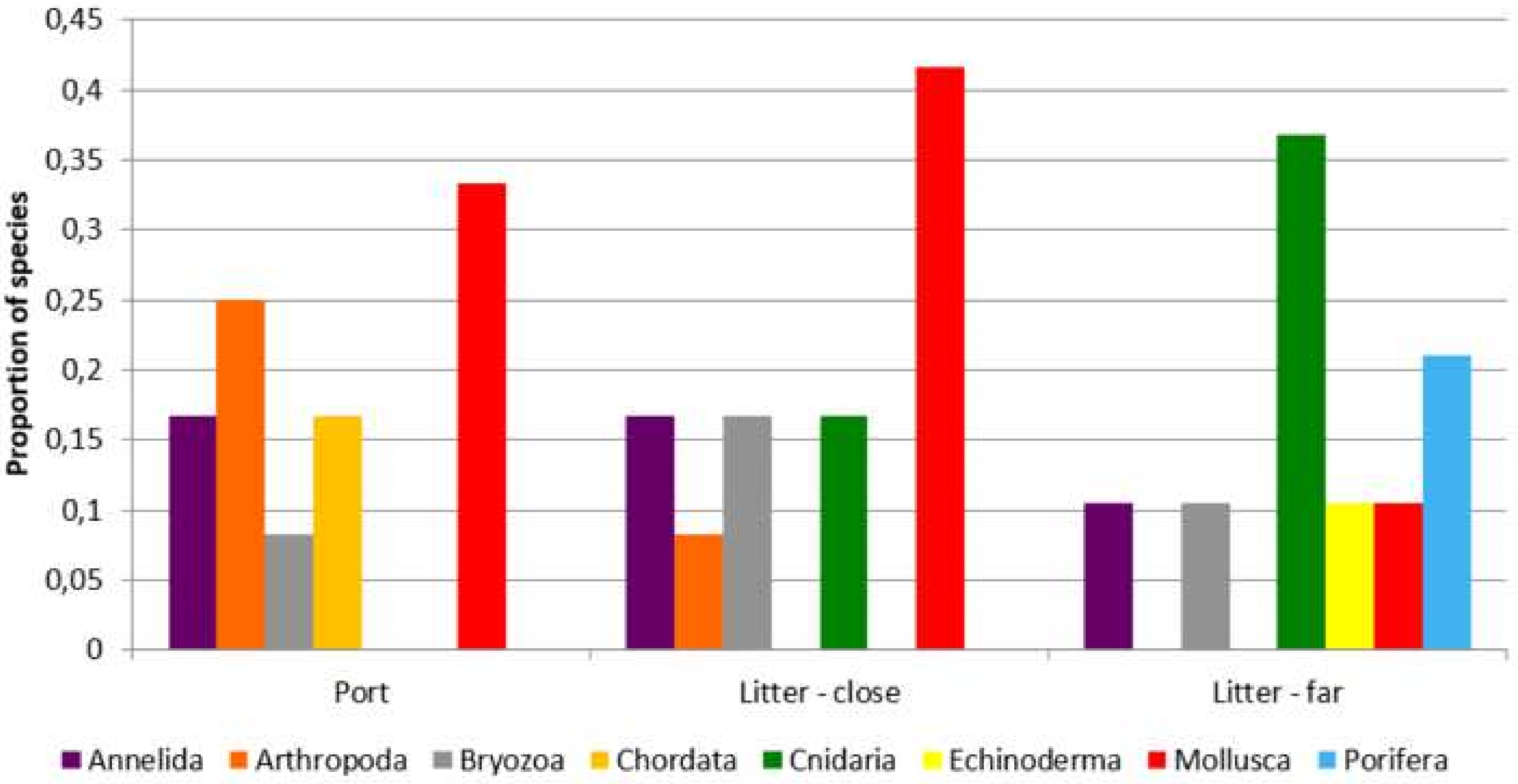
0,45
0,4
0,35
0,3
0,25
0,2
0,15
0,1
0,05
0
Proportion of species
Port
Litter - close
Litter - far
Annelida
Arthropoda
Bryozoa
Chordata
Cnidaria
Echinoderma
Mollusca
Porifera

S1 Table

Click here to access/download
Supporting Information
S1 Table.docx

S2 Table

Click here to access/download
Supporting Information
S2 Table.docx

S3 Table

Click here to access/download
Supporting Information
S3 Table.docx
