# Supplementary material for: Environmental DNA from plastic and textile marine litter detects exotic and nuisance species nearby ports
Source: PLoS One. 2020 Jun 18;15(6):e0228811. doi: 10.1371/journal.pone.0228811 (PMC7302909; doi:10.1371/journal.pone.0228811)
Supplement: Attachment — Submitted filename: Response to Reviewers.docx [file pone.0228811.s006.docx]

**Response to reviewers**

**Updated requirements**

The title should be adjusted to be more specific. For instance, the title should indicate what it is that is being dispersed (note the section of the title "secondary species dispersal". Dispersal of what in particular?) Additionally, noting that the technique picked up some species that are not being dispersed by plastic (i.e. cattle), the title should specify the e-DNA aspect of the analysis.

**Authors: OK, done. A new adjusted title was added: “Environmental DNA from plastic and textile marine litter detects exotic and nuisance species nearby ports.”**

The calculated means in the analysis should be provided alongside an estimation of error such as standard error or standard deviation. Similarly, discussion of estimation of error should occur alongside discussion of the means.

**Authors: OK, done. Values for standard deviation for the comparison of species presence over textile and plastic surfaces was added for Bryozoa and Florideophyceae groups, which show statistically significant differences.**

While revising your submission, please upload your figure files to the Preflight Analysis and Conversion Engine (PACE) digital diagnostic tool.

**Authors: OK, done. Figures were modified following PACE criteria.**

**Journal Requirements:**

In your Methods section, please provide additional information regarding the permits you obtained for the work. Please ensure you have included the full name of the authority that approved the field site access and, if no permits were required, a brief statement explaining why.

**Authors: OK done.**

We note that Figure 1 in your submission contain [map/satellite] images which may be copyrighted. All PLOS content is published under the Creative Commons Attribution License (CC BY 4.0), which means that the manuscript, images, and Supporting Information files will be freely available online, and any third party is permitted to access, download, copy, distribute, and use these materials in any way, even commercially, with proper attribution. For these reasons, we cannot publish previously copyrighted maps or satellite images created using proprietary data, such as Google software (Google Maps, Street View, and Earth).

We require you to either (1) present written permission from the copyright holder to publish these figures specifically under the CC BY 4.0 license, or (2) remove the figures from your submission:

**Authors: OK done. A new replacement figure was done, obtained from Natural Earth (public domain):** [**http://www.naturalearthdata.com/**](http://www.naturalearthdata.com/)

**Reviewer #1**:

-English revision of the text is needed.

**Authors: OK done.**

-Dinophyceae has to be revised along the text.

**Authors: OK done.**

-The authors used the primers targeting the COI region of metazoan (a taxon barcode for most animal taxa). These primers were targeting a barcode region that is specific for animal phyla (Leray et al. 2013). How this can fit with the mismatch and amplification of target mitochondrial regions of algae such as Dinophyceae and Rhodophyta? The authors should show the alignment of COI primer sequences with microalgae and macroalgae taxa COI sequences demonstrating the universal versatility of these COI primers designed for animal taxa.

**Authors: Added to discussion. Cytochrome Oxidase I (COI) has been confirmed as an effective tool for the barcoding and identification of algae and Dinophyte species (Robba et al., 2006; Le Gall & Saunders, 2010; Stern et al., 2010; Kogame et al., 2017).**

-The retrieving of epi-flora and fauna on marine debris through the COI metabarcoding method did not demonstrate that eDNA were from alive individuals or dead/fragment organisms. As also the authors commented, a new approach of investigating the eDNA/eRNA metabarcode ratio could be more adequate in order to explain the effective risk that NIS or invasive biota can colonize new areas. This assumption can be better expressed in the text of the manuscript with an alert of what you are retrieving on debris fragments in the marine environment: dead or alive, fragment or entire individual/larvae, eggs, propaguli. Instead, it is more likely that unicellular organisms such as marine microalgae can survive attached to plastics or debris.

**Authors: Added to discussion. It is true that most likely unicellular species can survive attached to marine litter, however, many species that were detected in this study were millimetric organisms (such as polyps) or species containing planktonic larval stages that can adhere to litter items when floating as plankton and employ its surface in the adult phase (due to their size commonly smaller than 1mm).**

- The NMDS is not clear; the authors can represent the distance correlation among the sites better.

**Authors: OK, the NMDS results were better explained.**

**Reviewer #2**

- Marine litter is not a problem of "nowadays": it exists from the beginning of the oceans. Probably Man inserted novelties (materials and /or quantities) in this framework, and really Authors probably speak about the only "Man derived litter"

**Authors: OK, done.**

- Ports could be considered sink as well as source areas for moving propagules. What is found on beaches, however, is more likely arrived material than ready-to be exported. Also in this case Authors should be careful in considerations and conclusions and I suggest a robust use of literature to justify such a point of view (they analyzed what arrived on the beaches, why they propose those materials are originated in the same area and ready to start?), or to re-examine the manuscript discussion from a different point of view.

**Authors: This is an interesting point. We added it to the discussion.**

**In some cases, ports can be sinks as well as donors of species. However, the received species can be newly transferred to neighboring areas after arriving into a port. In the case of the port of Gijon, marine currents flow eastwards in winter, so the port could be sink of litter coming from the west. However, this litter and species attached to it could be afterwards spread to the east from the port, which is why we consider it as the origin of the sampled litter items, as all the beaches are located eastwards the port.**

**Reviewer #3**

- I commend the authors on tackling a timely problem with a molecular tool of emerging significance for documenting invasive species. The statistical data analysis and interpretation were relatively thorough, sound, and appropriate. The data support their conclusions. Authors appropriately addressed limitations of the study and offered good suggestions for future work. The main concern was the lack of detail regarding sample collection/selection. By providing this critical information, the manuscript will be strengthened.

**Authors: OK, missing information about sample collection was added. The whole beach surfaces were sampled and all litter pieces bigger than 5cm were taken. No transects nor quadrats were employed, as all the surface was analyzed and every litter piece was collected).**

-Also, an expanded discussion on the different types of organisms found near and far from the port, their life histories, and additional explanation for these observations would greatly improve the manuscript.

**Authors: OK, done. Information about planktonic larval stages, and their size was added, also including information about adult benthic phases and their size (small enough to attach to litter items bigger than 5cm). Also, the tolerance to pollution and the presence/absence of some species on litter pieces from beaches that are close to the port is mentioned.**

-Finally, additional review of grammar, vocabulary, and sentence structure would benefit the manuscript.

**Authors: OK, done.**

-Line 14: Change “nowadays” to “currently” and “important” to “significant”

**Authors: OK, done.**

-Lines 17-18: Change to “endangering autochthonous communities”

**Authors: OK, done.**

-Line 20: Change “biofilm” to “biofilms”

**Authors: OK, done.**

-Lines 43-44: Although likely, there are no known studies documenting health impacts on humans. If I am mistaken, please provide a citation for this statement.

**Authors: information added. Marine litter can cause impact on human health. The degradation of litter generates microplastics that can be transferred along the food web and affect humans that consume them indirectly via contaminated marine food; this exposure to microplastics can result in chromosome alteration which lead to infertility, obesity and cancer.**

-Line 55: Add “a” between “provide” and “surface”

**Authors: OK, done.**

-Lines 58-59: Commas are needed after “with” and “on”. Suggest removing “to” and replacing with “and”.

**Authors: OK, done.**

-Line 64: Please further specify the impacts of Caulerpa taxifolia

**Authors: Example changed from *Caulerpa taxifolia* to *Undaria pinnatifida*, which forms dense mats and obstructs light inhibiting shellfish growth.**

-Line 77: Suggest removing “Nowadays” and replacing with “More recently”

**Authors: OK, done.**

-Lines 90-93: This sentence is not clear, please resolve

**Authors: OK, done.**

-Line 95: Replace “on” with “of”

**Authors: OK, done.**

Line 97: Please explain how you derived the 1.5%

**Authors: This percentage was a rough estimate of the surface of the litter, which was calculated considering the average size of the sampled items. However, we have removed this data since it can lead to confusion.**

-Change “Material” to “Materials”

**Authors: OK, done.**

-Lines 108-110: As written, this sentence is unclear. Please clarify.

**Authors: OK, done.**

-Please include more detail on sampling methods. Were samples collected along a transect? Using a quadrat? Randomly or haphazardly?
What was the outside temperature? Were samples placed on ice?

**Authors: OK, done. No transect nor quadrats were employed, as all the beach surface was considered, by collecting every litter piece that was bigger than 5cm. Litter items containing biofilm were collected in sterile tubes and stored in ethanol. The outside temperature was below 10 °C as sampling was done in winter.**

-Line 135: Replace “employed” with “used” and replace “take out” with “sample” or “collect”

**Authors: OK, done**

-Line 139: Replace “take out” with “remove”

**Authors: OK, done**

-Line 165: Please specify which version of QIIME was used
**Authors: OK, done.**

-Line 224: Can you please explain what the term “sanitary pad” refers to in this study? In some places, this term is used to describe a woman’s menstrual product. Is that what it is describing here? I just want to clarify as these are typically made of a combination of cotton and plastic.

**Authors: OK done. The term “sanitary pad” refers to compresses. It is true that these are typically made of cotton and plastic, however, eDNA for extraction was only taken from the textile part, which was the one with macroscopically observable biofilm.**

-Lines 232-234: This sentence reads as out of place since the biota are discussed in the next section.

**Authors: OK, done. The sentence and the figure it refers were removed, as they are discussed in the next section.**

-Lines 240-246: This paragraph might be better suited for the discussion.

**Authors: OK, done.**

-Line 263: Please change the symbol to a percentage %

**Authors: OK, done.**

-Line 276: Change “assignation” to “assignment,”

**Authors: OK, done.**

-Lines 309-310: Suggest changing “; likely runoffs” to “, where it is likely that runoff”

**Authors: OK, done.**

-Line 316: Replace “bigger amount” with “higher number”

**Authors: OK, done.**

-Table 3: As a suggestion, the data may be presented in a more digestible format as a bar chart or pie chart paired with an inset of a map of the bay. I find myself having to keep referring to the map.

**Authors: OK, done. A new figure has been done, representing results in a more visual way (Figure 5).**

-Figures 4A and 4B: Need percentages or species numbers labels associated with the pie charts

**Authors: OK, done.**

-Line 351: Suggest replacing “nowadays” with “currently”

**Authors: OK, done.**

-Line 358: Insert “a” after “from”

**Authors: OK, done.**

-Line 379: Could you please provide a bit more detail regarding the published data from the port of Gijon (e.g. briefly describe what types of samples were collected and how they were analyzed.)

**Authors: OK, done. These samples were taken from three different sites in the port of Gijon; one near the port mouth, one in the inner section and another one half way between these two. Approximately 200m^2^ of artificial port structures were sampled in each site, where visual inspections were done prior to sampling in order to detect phenotypically different organisms and identify as many different species as possible, targeting all macroscopic biota inhabiting the port of Gijon.**


-Line 475: Suggest replacing “big” with “large”

**Authors: OK, done.**

**Reviewer #4:**

-There needs to be a clarification early on about what types of textiles these are, and how textiles fit in as a subset of marine debris. In reality, many textiles these days are almost fully synthetic, and so are themselves made of plastic. Especially sanitary pads are most likely majority plastic. If the researchers have access to FTIR spectroscopy, it would be a great addition to the paper to know if these clothing items were in fact 100% cotton, or wool, or mostly synthetic, and how that changed the biofilm composition.

**Authors: OK done. Information about what textile litter is considered was added. It is true that sanitary pads are most likely majority plastic, however, only their textile part was employed for the extraction of DNA from biofilm, as the plastic part of sanitary pads did not show visible biofilm. FTIR spectroscopy would be a great addition to improve the results, unfortunately we have no access to it.**

-Similarly, when fishing gear is discussed, it should be clarified if it was all plastic and synthetic fiber or if there was metal gear attached.

**Authors: OK, done.**

-A careful proofreading is recommended.

**Authors: OK, done**

-Line 165-169: I do not believe it is proper to put the scripts used, but rather explain what that script did.

**Authors: OK, done**

Similar to 180-183, the actual website link does not need to be in the paper.

**Authors: OK, done**

-Figure 3: Please explain more in the Results section. Why is Penarubbia apart?
**Authors: OK, done. Peñarrubia is the only pebble beach that is outside the city of Gijon, thus, it is not cleaned in winter. Apart from this, it is not a sheltered beach as all the others are. This can be the cause of differentiation from other beaches.**

-Table 2: Explain the Code column in Table heading. What are units on last three columns?

**Authors: OK, done. Codification is explained as: initial letter of the beach; P for plastic, T for textiles. For example; Ñ-P1 is the first plastic item from La Ñora beach. Units for the last three columns are number of sequences.**

-Lines 308-311: There is also the marine insect Halobates that is known to lay its eggs on marine debris and has been shown to benefit from the increase in marine debris in recent years (Goldstein et al. 2012). If the DNA was from Halobates or their near relatives, it should not be excluded.

**Authors: In our case, DNA was not from Halobates, however, we added this information as probably other insects could also benefit from marine litter.**

-Table 4: a clarification between alien, invasive, and non-native species needs to be made early in the paper, or it needs to be clear that these are being used interchangeably. Are these of varying severities?

**Authors: Each term means a different thing, so we have modified the manuscript in order to make it easier to understand. We now employ the following terms:**

**Non-indigenous species (NIS): these are species that are not native from the study area, but are not considered stablished yet (early phases of introduction, low number of organisms)**

**Alien invasive species (AIS): these are exotic species that have successfully invaded the study area and are already stablished (and causing severe problems)**

The other things in this table that are pathogenic need to be discussed more thoroughly.

**Authors: OK done. We added information to discussion about previous studies that also found marine litter to be a vector for potentially harmful species. We have also pointed out the high proportion of these species that appear in textile litter (45%) and discussed these data.**

-Line 501-504: Why did you not use RNA if it would have been more effective?

**Authors: It is true that eRNA could distinguish between alive and dead organisms, however, some disadvantages related to eRNA are overrepresentation of organisms with complex genomes and numerous copies of transcriptionally active marker genes (Gong et al., 2013), or different artifacts that can occur during RNA processing and PCR amplification (Laroche et al., 2017). This is why eDNA was employed in this study, although it is not possible to define if the detected species were alive organisms fouling marine litter.**

**Reviewer #5:**

-A lot of unnecessary verbiage introduces the article, which should start at lines 87-88 ("There are solid studies ...", but rather read, "There is an increasing number of studies...", not "solid").

**Authors: OK done.**

All of the introductory material is unnecessary: there is no need to introduce readers to the basics of invasion science, marine litter, or DNA analyses, no more than one would introduce a reader to elementary descriptions of community or ecosystem ecology in a study on, for example, intertidal species). The paper should be edited by a native English speaker.

**Authors: We understand that the information given in the introduction can be unnecessary for expert readers, however we do think that it may be useful for non-expert readers, to guarantee a proper understanding of the paper and its results, so as the rest of reviewers consider it correct, we will keep this information in the introduction.**

On a technical note, the "hit" of Squamamoeba japonica should be discarded out right -- it is not "difficult to explain" (as the authors write) -- it's a transparent artifact.

**Authors: OK done.**

**Reviewer #6**

General Comments:

I encourage the authors to reassess how they use biofilm. The term “biofilm” is usually reserved to characterize microorganisms on substrata. Here, it seems the authors use it to refer to all organisms found on your plastics, both micro and macro. A term such as “epiplastic biota” or “biofouling” might be more appropriate. Building upon this point, it would be helpful for the authors to include a selection of photographs of the items observed, so the reader can get a sense of the biofouling present.

**Authors: OK done. A new figure has been included for the visualization of the biofilm on litter. As it can be seen there, no visible macroscopic biota was found in any litter item, this is why the term biofilm is employed. However, this comment was considered and the term biofouling is now employed to refer to the communities (including microscopic and macroscopic species) detected on the litter.**

Once you introduce the concept and process of environmental DNA in the Introduction, I would use the term eDNA rather than environmental DNA for the remainder of the manuscript. eDNA is common terminology at this point.

**Authors: OK done**

An important point, which I touch on more in my specific comments, is that the authors need to make it clear in the methods section how many items were actually sampled for their biofouling and how many were then assessed genetically.

**Authors: OK done.**

Specific Comments:

-P 1, l. 20: Usually biofilm refers to the microorganisms attached rather than macrobiota, like bryozoans. If you mean both micro- and macroorgranisms, I suggest using the terms “epiplastic biota”.

**Authors: OK done. Instead of employing the term “epiplastic biota” which would only refer to plastic litter (and we want to emphasize the importance of textile litter) we changed to the term to “biofouling”.**

P 1, ll. 26-29: Current sentence structure is a bit confusing. Do you mean to say that beaches closer to ports were more similar to their nearby port than beaches farther away?

**Authors: OK done. Sentence structure was modified to make it more understandable that biota from beaches closer to the port was more similar to their nearby port than the one from beaches farther away.**

P 2, l. 36: “are” should be “have been”

**Authors: OK done**

P2, ll. 37-40: Consider changing this sentence to: “Human activities such as agriculture, fisheries, or industry, overexploit natural resources, and as a result rates of species extinction are now 100 to 1000 times higher than prior to human presence.”

**Authors: OK done**

P2 l. 47: Insert comma between “industries” and “such”

**Authors: OK done**

P2 l. 48: remove comma after “from nets”

**Authors: OK done**

P2 l. 49: Remove “an average of”

**Authors: OK done**

P2 l. 52: Replace “leading” to “and lead”

**Authors: OK done**

P2 ll. 53-54: Reword this sentence. Consider wording like, “The role of marine debris as a dispersal vector of invasive organisms is of special concern.”

**Authors: OK done**

P3 ll. 87-88: I would consider referencing the extensive work done on invasive species transport following the 2011 Japanese tsunami, as this research presented the most extraordinary example of marine debris invasive species dispersal. Suggested texts include, but are not limited to, Carlton et al. 2017, Science; Simkanin et al. 2018, Global Ecology and Biogeography; and Carlton et al. 2018, Aquatic Invasions.

**Authors: OK done.**

P 3 ll. 88-90: The wording of this sentence is confusing. Consider something like, “…however, to our knowledge no research has examined the role of textile litter as a vector.”

**Authors: OK done**

P 3 ll. 87-90: Following this sentence, you need to define textile litter for the reader.

**Authors: OK done**

P 3 ll. 90-93: Revise this sentence for conciseness and clarity.

**Authors: OK done**

P 3 l. 94: “was” should be “were”

**Authors: OK done**

P 3 l. 95: “on” should be “of”

**Authors: OK done**

P 4 l. 96-99: The wording of this sentence is confusing. Please revise.

**Authors: OK done**

P 4 ll. 99-100: Consider reworking this sentence to something like, “Fauna found on litter were compared to fauna growing on structures in nearby ports to assess faunal origin.”

**Authors: OK done**

P 4 l. 106: Remove “at the”

**Authors: OK done**

P 4 ll. 108-110: There are a few important ideas in this sentence, but the current structure is confusing. Consider changing to, “Beaches east of Gijon port were selected for our study for two reasons: (1) Gijon is a potential donor of marine invasive species; (2) in the winter, at the time of the study, dominant currents flow eastward along the coast, likely depositing debris from Gijon on beaches to the east.”

**Authors: OK done**

P 4 l. 118: surface area?

**Authors: the sentence was modified.**

P 5 l. 135: Replace “take out” with “remove”

**Authors: OK done**

P 5 l. 136: Insert comma between “Sterile” and “DNA/RNA”, unless this is the manufactured name of a product.

**Authors: OK done**

P5 ll. 139-140 How did you maintain sterility of the Stomacher?

**Authors: It was cleaned after each use with different samples to maintain sterility.**

P 5 l. 142: Replace “liquid excess” with “excess liquid”

**Authors: OK done**

P 5 l. 143: Remove “the rest of”

**Authors: OK done**

P 7 l. 198-199: This sentence could use further clarification. What do you mean by environmental DNA and assignment artifacts were excluded from the analysis?

**Authors: OK done. Assignment artifacts refer to singletons and wrong species assignments due to the scarcity of reference sequences for certain taxa on NCBI database.**

P 7 l. 212: Insert “area” after “surface”

**Authors: OK done**

P 7 l. 213: Replace “in” with “on”

**Authors: OK done**

P 7 l. 215: Insert “area” after “surface”

**Authors: OK done**

P 7 l. 216: units of measurement are missing after “18.6”

**Authors: OK done**

P 7 l. 216: Replace semicolon with period, making the following clause a separate sentence

**Authors: OK done**

P 7 Table 1: “Beach surface” should be “beach surface area”

**Authors: OK done**

P 8 ll. 224-225: Reword this sentence or break into multiple sentences.

**Authors: OK done**

P 8 ll. 226-227: Make sure that you are complying with PLoSONE’s formatting standards for reporting statistical results in text. Usually this would be presented as χ 40 2 : 837.94; p = 6.31x10-150; Monte Carlo p= 0.0001).

**Authors: OK done.**

P 8 ll. 232-233: Change “being Penarrubia and Cagonera beaches the most similar ones” to “with Penarrubia and Cagonera beaches being the most similar”

**Authors: This sentence was removed as it is discussed in the next section.**

P 8 l. 241: Remove “punctual”

**Authors: OK done**

P 8 l. 252: Replace “of the former” with “from those rich in textiles”

**Authors: OK done**

P 8 l. 252: Replace “apart” with “isolated”

**Authors: OK done**

P 9 l. 259: Replace “from” with “with”

**Authors: OK done**

P 9 l. 262: 16 types of what?

**Authors: modified to “16 litter items”**

P 9 ll. 261-267: I am confused about the number of samples that were surveyed for eDNA sequencing versus those that were actually used in the genetic analysis. This should be further clarified in the methods section. I was under the impression that the 1000+ samples collected on the beaches were sampled, but now I realize that might not be the case. Please clarify.

**Authors: The sentence was modified in order to clarify that** **from all the litter items that were found (more than 1000), only the ones containing visible biofilm (16 items) were stored for eDNA sequencing.**

P 10 l. 289: Remove “still”

**Authors: OK done**

P 10 l. 301: Replace “After all the process,” with “In total,”

**Authors: OK done**

P 10 l. 303: Insert comma between “organisms” and “but”

**Authors: OK done**

P 11 l. 309: “Runoff” rather than “runoffs”

**Authors: OK done**

P 11 l. 310: Insert comma after “insects”

**Authors: OK done**

P 11 l. 316: Replace “bigger” with “greater”

**Authors: OK done**

P 12 l. 336: Replace “in” with “on”

**Authors: OK done**

P 12 l. 341: Replace “DNA from” with “DNA, as”

**Authors: OK done**

P 13 Table 4: Use a more descriptive term in place of “Issue”

**Authors: OK done**

P 15 l. 407: Replace “security” with “biosecurity”

**Authors: OK done**

P 15 l. 417: Make textile plural

**Authors: OK done**

P 15 l. 421: Remove “a priori”

**Authors: OK done**

P 15 ll. 420-423: This is a run-on sentence. Please consider breaking this into two sentences.

**Authors: OK done**

P 15 l. 423: Remove “Nowadays focused on massive plastic debris,” and be sentence with “Future”

**Authors: OK done**

P 15 l. 431: Remove “this”

**Authors: OK done**

P 15 l. 432: Remove comma after citation 36

**Authors: OK done**

P 16 l. 437: Replace comma with semicolon

**Authors: OK done**

P 16 l. 437: Remove “could be potentially dangerous in these areas, as it”.

**Authors: OK done**

P 16 l. 445: Remove comma

**Authors: OK done**

P 16 l. 458: Replace “rests” with “remains”

**Authors: OK done**

P 16 ll. 457-459: This is an important point. Consider concluding this paragraph with a sentence about the implications of this result. For example, this means that eDNA samples do not guarantee detection of live organisms.

**Authors: OK done (added to discussion).**

P 17 l. 478: Replace comma with semicolon

**Authors: OK done**

P 17 ll. 492-495: This is an important point. I would add a sentence to the end of this paragraph, which states that assignment artifacts are possible for other aquatic and marine organisms that are not well defined.

**Authors: OK done.**

P 17 l. 498: Awkward wording. Consider replacing the first comma with a semicolon, following by,“…that is, we cannot tell if the presence detected reflect an ability to colonize new material.”

**Authors: OK done**

P 18 l. 512: Replace “to the” with “with”

**Authors: OK done**

P 18 l. 513: Replace “would be” with “is”

**Authors: OK done**

P 18 l. 515: Replace “would be” with “is”

**Authors: OK done**

P 19 l. 536: Capitalize “pollution bulletin”

**Authors: OK done**

References – Be sure that your formatting of journal names is consistent. Sometimes their italicized and sometimes not. The same goes for the volume and page numbers.

**Authors: OK done**
